# Fillable and unfillable gaps in plant transcriptome under field and controlled environments

**DOI:** 10.1111/pce.14367

**Published:** 2022-06-21

**Authors:** Yoichi Hashida, Ayumi Tezuka, Yasuyuki Nomura, Mari Kamitani, Makoto Kashima, Yuko Kurita, Atsushi J. Nagano

**Affiliations:** ^1^ Faculty of Agriculture Takasaki University of Health and Welfare Takasaki Gunma Japan; ^2^ Research Institute for Food and Agriculture Ryukoku University Otsu Shiga Japan; ^3^ Faculty of Agriculture Ryukoku University Otsu Shiga Japan; ^4^ College of Science and Engineering Aoyama Gakuin University Sagamihara Kanagawa Japan; ^5^ Institute for Advanced Biosciences Keio University Tsuruoka Yamagata Japan

**Keywords:** biotic and abiotic stress, circadian clock, field, growth chamber, rice, RNA‐Seq, sugar metabolism, transcriptome

## Abstract

The differences between plants grown in field and in controlled environments have long been recognized. However, few studies have addressed the underlying molecular mechanisms. To evaluate plant responses to fluctuating environments using laboratory equipment, we developed SmartGC, a high‐performance growth chamber that reproduces the fluctuating irradiance, temperature and humidity of field environments. We analysed massive transcriptome data of rice plants grown under field and SmartGC conditions to clarify the differences in plant responses to field and controlled environments. Rice transcriptome dynamics in SmartGC mimicked those in the field, particularly during the morning and evening but those in conventional growth chamber conditions did not. Further analysis revealed that fluctuation of irradiance affects transcriptome dynamics in the morning and evening, while fluctuation of temperature affects transcriptome dynamics only in the morning. We found upregulation of genes related to biotic and abiotic stress, and their expression was affected by environmental factors that cannot be mimicked by SmartGC. Our results reveal fillable and unfillable gaps in the transcriptomes of rice grown in field and controlled environments and can accelerate the understanding of plant responses to field environments for both basic biology and agricultural applications.

## Summary Statement

Diurnal transcriptome dynamics of rice grown in fluctuating field and controlled environments are different mainly in the morning and evening, which are affected by light and temperature in the morning, while only by light in the evening. We also found field‐specific gene expression.

## 1 INTRODUCTION

To optimize agricultural crop productivity and understand plant behaviour in natural environments, knowledge of plant responses to fluctuating field environments is essential. Numerous studies conducted in controlled environments, such as growth chambers and greenhouses, have facilitated the understanding of plant responses to environmental stimuli. However, such responses are sometimes different from those in controlled environments (Annunziata et al., 2017, 2018; Dantas et al., 2021; Matsubara, 2018; Matsuzaki et al., 2015; Nagano et al., 2012; Poorter et al., 2016; Song et al., 2018) due to differences between the two environments. Field environments experience daily fluctuations and gradual changes, particularly around dawn and dusk, whereas controlled environments usually fluctuate quickly and regularly between fixed (i.e., square‐wave) conditions, which are constant during the day and night, and abruptly transition at dawn and dusk. Light quality, such as red light to far‐red light ratio and the presence of ultraviolet‐B light, also varies between field and controlled environments. Additionally, plants in the field experience abiotic and biotic stresses, such as wind, precipitation, and insect and pathogen attacks. Such factors make it difficult to apply knowledge obtained from laboratory studies to the field studies in plant science.

To reveal plant responses to fluctuating field environments, field and laboratory studies have attempted to address the differences between the two settings. One approach involves transcriptome analysis of field‐grown plants (Dantas et al., 2021; Iwayama et al., 2017; Kashima et al., 2021; Matsuzaki et al., 2015; Nagano et al., 2012, 2019; Takehisa & Sato, 2019; Zaidem et al., 2019). We previously developed a statistical model that predicts the transcriptome dynamics of rice leaves in the field using meteorological data (Nagano et al., 2012). The modelling approach provides valuable information about plant responses to the field environment, although the detailed mechanism still requires examination under laboratory conditions. Another approach is to mimic the field environment using laboratory equipment. Studies using this approach have clarified the characteristics of photosynthesis under fluctuating light (Alter et al., 2012; Kaiser et al., 2018; Matsubara, 2018; Niedermaier et al., 2020; Schneider et al., 2019; Tanaka et al., 2019; Vialet‐Chabrand et al., 2017; Yamori, 2016), successfully mimicked primary metabolism of Arabidopsis leaves (Annunziata et al., 2017, 2018), and determined the expression patterns of the Arabidopsis florigen gene, *FLOWERING LOCUS T* (*FT*) (Song et al., 2018) in field environments.

Previous studies have clarified the characteristics of plant responses to fluctuating field environments. However, a comprehensive understanding of the differences between plants grown in field and in controlled environments is still lacking. Therefore, we developed SmartGC, a high‐performance growth chamber that can reproduce fluctuating field environments, to compare plant responses to field and controlled environments. By analysing massive transcriptome data of rice plants grown under field and SmartGC conditions, we revealed fillable and unfillable gaps in plant responses to field and controlled environments.

## 2 MATERIALS AND METHODS

### 2.1 Plant materials and growth conditions

In this study, we developed SmartGC, a high‐performance growth chamber (Supporting Information: Figure S1a–d). SmartGC is composed of two parts: a growth chamber (LPH‐240SP, Nippon Medical & Chemical Instruments Co., Ltd.) (for controlling temperature and relative humidity) and a Heliospectra L4A LED light source (Heliospectra) (for controlling light). Both parts have been customized to be controlled simultaneously by one computer, and they are scheduled to function for more than 24 h. The light source can independently control seven types of LEDs (violet to far‐red) with a 1‐s resolution, but it does not include UV‐A and UV‐B. The spectrum of the light source is shown in Supporting Information: Figure S2a,b. The output value of each LED can be set to 0 or 1 in increments from 15 to 1000 sv (set value). Temperature and relative humidity were set to 15–45°C and 50%–80%, respectively, at a 1‐min resolution. SmartGC can record temperature and relative humidity every minute. Although the light source can control seven types of LEDs independently, we set the output of all LEDs to the same value for each setting.

**Figure 1:**
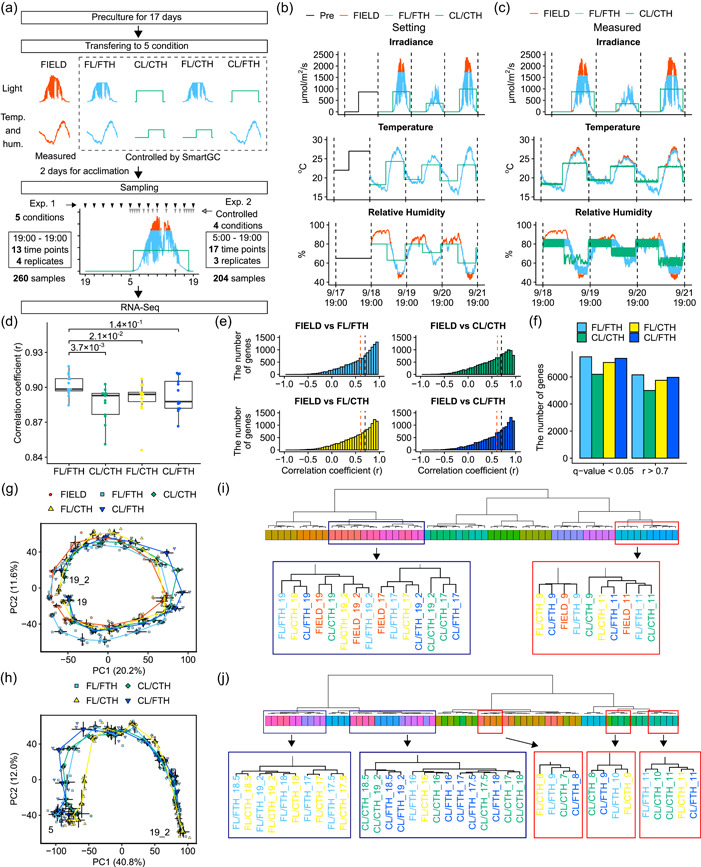
Transcriptome dynamics of rice leaves in the field are mimicked under simulated field environments in SmartGC. (a) Experimental design of this study. (b) Irradiance, air temperature and relative humidity in the preculture, FIELD, FL/FTH and CL/CTH conditions. The unit of irradiance is photon flux density (PFD) defined over 380–780 nm. (c) Measured values of irradiance, air temperature and relative humidity in FIELD, FL/FTH and CL/CTH. (d) Boxplots showing pairwise Pearson's correlation coefficients (*r*) of transcriptomes between FIELD and the other conditions at each time‐point. Adjusted p‐values of Wilcoxson rank‐sum test between FIELD versus FL/FTH and FIELD versus CL/CTH, FL/CTH and CL/FTH are shown. Each point shows the mean value of four replicates. (e) Histogram of pairwise Pearson's correlation coefficient of expression levels of each gene between FIELD and the other conditions. (f) A bar graph showing the number of genes with *r* > .7 and *q*‐value < 0.05 in (e). (g) and (h) Principal component analysis (PCA) of transcriptomes in (g) Experiment_1 and (h) Experiment_2. The percentages of total variance represented by principal component 1 (PC1) and principal component 2 (PC2) are shown in parentheses. Each point shows the mean value of the four replicates; error bars indicate the standard errors of PC1 and PC2. Points were connected by lines according to the time‐point at each condition. Numbers within the frame indicate the start and end times. 19_2 indicates the time‐point 24 h after the start of sampling day at 19:00. (i) and (j) Hierarchical cluster dendrograms of the transcriptomes in each condition and time‐point in (i) Experiment_1 (*n* = 4) and (j) Experiment_2 (*n* = 3). Same‐coloured panels in the dendrograms indicate the same sampling time.

A common japonica rice (*Oryza sativa* L.) cultivar, Nipponbare, was used in all the experiments in this study. Seeds were sterilized in a 2.5% (v/v) sodium hypochlorite solution for 30 min and then soaked in water at 30°C for 3 days. Germinated seeds were sown in a cell tray filled with nursery soil (N:P_2_O_5_:K_2_O = 0.6:1.2:1.0 g/kg). Plants were grown in SmartGC for 17 days with a 14 h photoperiod and an irradiance level of 867 µmol photon m^−2^ s^−1^ (photon flux density [PFD] of 380–780 nm) 30 cm from the light source, which corresponds to the height of the middle part of the rice leaves used for sampling, by setting an output value of 500 sv (Figure 1a,b, Supporting Information: Figure S1b). The daily light integral (DLI) or the number of photosynthetically active photons (400–700 nm) accumulated in a square metre over the course of a day, was 38mol photons m^−2^ day^−1^. Plants were then transferred to each condition as follows (Figure 1a):

1. Field condition (FIELD). We chose a site at Ryukoku University, Otsu, Japan (34°57′43.4″N, 135°56′22.6″E) for the experiment (Supporting Information: Figure S1e,f). Plants were transferred at 19:00 on 18 September 2017. During the field experiment, temperature, relative humidity and irradiance were measured every minute (Figure 1b). Temperature and relative humidity were measured using THMchip thermo‐hygrometers (THM10‐TH, FUJIFILM Wako Pure Chemical Corporation) which were set in an aspirated radiation shield (Okada & Nakamura, 2010). Irradiance was measured using a quantum metre (LA‐105, Nippon Medical & Chemical Instruments Co., Ltd.). As we could not obtain irradiance from 16:00 to 17:31 on 19 September 2017 (i.e., 21–22 h and 31 min after transferring plants to the field), we regarded the change in irradiance during this time as a linear decrease and used the calculated value of irradiance for further experiments (Figure 1b,c). The spectrum and red (655–665 nm) to far‐red (730–740 nm) (R:FR) ratio of irradiance is shown in Supporting Information: Figure S2a–c, respectively. The R:FR ratio ranged from 1.1 to 1.6. The DLI was 30, 13 and 34 mol photons m^−2^ day^−1^ on the first, second and third days, respectively.
2. Fluctuating light, temperature and humidity (FL/FTH). Fluctuations in irradiance, temperature and relative humidity in the FIELD condition were simulated. Irradiance in the FIELD condition was simulated every minute by translating irradiance to the light source output using a calibration curve (Supporting Information: Figure S3). We measured the PFD of the output from 15, 100, 200, 300, 400, 500, 600, 700, 800, 900, 950 and 1000 sv for each LED and constructed a calibration curve using linear regression. Although plants can sense light below 1  µmol photon m^−2^ s^−1^, particularly through phytochrome A, and low light can affect gene expression (Seaton et al., 2018; Shinomura et al., 1996), we regarded irradiance in the FIELD with PFD < 1 as darkness and set the output value of the light source to zero. Since the lowest output value for turning on the light source was 15 sv, the output value during the day was set to 15 sv, as the irradiance in the FIELD was lower than that in SmartGC, with an output value of 15 sv (Figure 1b, Supporting Information: Figure S4a,b). The photoperiod was 12 h and 37 min, 12 h and 31 min and 12 h and 31 min on the first, second and third days, respectively. In addition, the highest output value for the light source was 1000 sv, and the output value during the day was set to 1000 sv when the irradiance in the FIELD was higher than that at an output value of 1000 sv in SmartGC (Figure 1b). Irradiance was measured every minute using a quantum metre (LA‐105) without plants, and the R:FR ratio of the irradiance was calculated (Figure 1c, Supporting Information: Figures S2c and S5a,b). The R:FR ratio ranged from 2.0 to 7.7. The DLI was 30, 13 and 32 mol photons m^−2^ day^−1^ on the first, second and third days, respectively. The temperature and relative humidity in the FIELD were simulated every 1 min. The relative humidity in the FL/FTH condition was less than 50% or more than 80%, so the humidity was set to 50% or 80% (Figure 1b). Temperature and relative humidity were logged every minute (Supporting Information: Figure S5c–f).
3. Constant light, temperature and humidity (CL/CTH). Irradiance, temperature and relative humidity were kept constant during the day and night. The time of dawn and dusk in Otsu, Shiga was that recorded by the National Astronomical Observatory of Japan. The photoperiod was 12 h and 16 min, 12 h and 13 min, and 12 h and 11 min on the first, second and third days, respectively. Each day, irradiance, temperature and relative humidity were set to constant values during the day and night, which corresponded to the average values in the field condition during the day and night, respectively. As we could not obtain the field data 72 h after sampling, the temperature and relative humidity after dusk (17:55–19:00) on the third day were set to the respective constant values at 19:00 in FIELD. The relative humidity in FIELD was less than 50% or more than 80%, so the humidity was set to 50% or 80% (Figure 1b). Irradiance was measured every minute using a quantum metre (LA‐105) without plants, and the R:FR ratio of the irradiance was calculated (Figure 1c, Supporting Information: Figures S2c and S5a,b). The R:FR ratio ranged from 3.1 to 5.2. The DLI was 33, 13 and 38 mol photons m^−2^ day^−1^ on the first, second and third days, respectively. Temperature and relative humidity were logged every minute (Supporting Information: Figure S4c–f).
4. Fluctuating light with constant temperature and humidity (FL/CTH). Light was set to the values in the FL/FTH condition, and the temperature and relative humidity were set to the values in the CL/CTH condition.
5. Constant light with fluctuating temperature and humidity (CL/FTH). Light was set to the same values as in the CL/CTH condition, and the temperature and relative humidity were set to the values of the FL/FTH condition.

After transferring the plants to each condition, they were acclimatized for 48 h. Sampling was conducted every 2 h for 24 h starting at 19:00 (13 times in total, Experiment_1) (Figure 1a, Supporting Information: Figures S4 and S6, Supporting Information: Table S1). Under each condition, the uppermost, fully expanded leaves (i.e., the fifth leaves) were sampled from all four plants per sampling point, frozen in liquid nitrogen and stored at −80°C for future use. At each sampling point, sampling was completed within 5 min.

Another sampling was conducted under FL/FTH, CL/CTH, FL/CTH and CL/FTH conditions to investigate plant responses around dawn and dusk in detail (Experiment_2). The experimental scheme was the same as that described above, except the sampling time‐points and the number of samples differed. Fifty‐eight to seventy‐two hours after transferring the plants to each of the conditions, three plants were sampled at each of the following time‐points: 5:00, 5:30, 6:00, 6:30, 7:00, 8:00, 9:00, 10:00, 11:00, 13:00, 15:00, 16:00, 17:00, 17:30, 18:00, 18:30 and 19:00 (17 times in total) (Figure 1a, Supporting Information: Figures S4 and S6, Supporting Information: Table S1).

### 2.2 RNA‐Seq analysis

The leaf samples were ground under cryogenic conditions using a Multi‐Beads Shocker (Yasui Kikai). Total RNA was extracted using the Maxwell 16 LEV Plant RNA Kit (Promega). RNA concentration was measured using the broad‐range Quant‐iT RNA Assay Kit (Thermo Fisher Scientific). RNA (500 ng) was used as the input of each sample for library preparation. Library preparation for RNA‐sequencing was conducted using Lasy‐Seq (Kamitani et al., 2019) version 0.9 or 1.0 (https://sites.google.com/view/lasy-seq/; Supporting Information: Figure S7). The library was sequenced using HiSeq. 2500 (Illumina) at Macrogen or Takara with single‐end sequencing lengths of 50bp or 100 bp, respectively.

All obtained reads were trimmed using Trimmomatic version 0.33 (Bolger et al., 2014) using the following parameters: TOPHRED33, ILLUMINACLIP:TruSeq. 3‐SE.fa:2:30:10, LEADING:19, TRAILING:19, SLIDINGWINDOW:30:20, AVGQUAL:20, MINLEN:40, indicating that reads with more than 39 nucleotides and average quality scores over 19 were reported. Then, the trimmed reads were mapped onto the reference sequences of the IRGSP‐1.0_transcript (Kawahara et al., 2013) and the virus reference sequences, which were composed of complete genome sequences of 7457 viruses obtained from NCBI GenBank (Kashima et al., 2021) using RSEM version 1.3.0 (B. Li & Deway, 2011) and Bowtie version 1.1.2 (Langmead et al., 2009) with default parameters.

The reads per million (rpm) were calculated using the nuclear‐encoded gene raw count data, excluding the genes encoding rRNA, as described by Kashima et al. (2021). In Experiments 1 and 2, 0.85–3.35 million and 1.11–4.27 million reads per sample were used for calculating rpm, respectively (Supporting Information: Figure S7a). A total of 12,741 genes in which the average number of reads was >10 in all Experiment_1 samples was used for the statistical analysis (Supporting Information: Figure S7b).

### 2.3 Inference of internal time using the molecular timetable method

We applied the molecular timetable method (Ueda et al., 2004) to the transcriptome data of Experiment_1 to infer the internal time of each sample, as described by Higashi et al. (2016). First, we selected time‐indicating genes whose expression indicated periodicity and high amplitude. To evaluate the periodicity, we prepared 1440 cosine curves, which had different peaks (0–24 h) measured at 1‐minute increments. We fitted the curves to the time‐course transcriptome data of FIELD in Experiment_1 (52 total samples) and calculated the correlation coefficient (*r*) to identify the best‐fitting cosine curve (Supporting Information: Figure S8a). The peak time of the best‐fitting curve was estimated as the peak time for each gene and was defined as the molecular peak time. Thus, the molecular peak time was estimated individually for each gene. Then, to analyse the amplitude, we calculated the average gene expression and standard deviation for each gene. The amplitude value (*a*) was calculated as the standard deviation divided by the average gene expression level (Supporting Information: Figure S8b). A total of 143 time‐indicating genes were selected according to the cut‐off values of *r*= 0.935 and *a*= 0.15 (Supporting Information: Table S2). The molecular peak time of the time‐indicating genes was covered throughout the day, which ensured the accurate estimation of internal time (Supporting Information: Figure S8c).

We normalized the expression level of each time‐indicating gene using the z‐score, which is defined as the value of the individual expression level minus the average expression level, divided by the standard deviation. We then plotted expression profiles composed of the molecular peak time and the normalized expression level for each sampling time (Supporting Information: Figure S8d). Finally, the internal time was estimated using a plotted expression profile. We prepared 1440 cosine curves (with each having 1‐min difference with respect to preceding one) and fitted them to the expression profiles. We identified the best‐fitting cosine curve, and the corresponding peak time was used to indicate the estimated internal time.

To validate the accuracy of inferring the internal time using the time‐indicating genes, we calculated the measurement noise as the standard deviation of the difference between the real and estimated expression of each time‐indicating gene. The measurement noise of each gene ranged from 91% to 100% (mean ± standard deviation: 99 ± 1%), indicating that 143 time‐indicating genes were sufficient for accurately estimating the internal time (Ueda et al., 2004).

### 2.4 Determination of starch and sucrose content

Starch and sucrose content were determined as described by Okamura et al. (2013).

### 2.5 Analysis of public microarray data

We used the microarray data previously analysed by Nagano et al. (2012). This data was available on the GEO website (https://www.ncbi.nlm.nih.gov/geo/; accession numbers: GSE36777 and GSE36595) and had already been normalized and log‐transformed. We used 96 samples of rice (cultivar: Norin 8) grown in paddy fields that were sampled in August 2009, 39–98 days after transplantation. We also used 16 samples of the same rice cultivar grown in a growth chamber, which were sampled at 2:00 and 14:00 at 30, 32, 34 and 36 days after sowing. Details about the samples and conditions in the experiment are described in the study by Nagano et al. (2012). The parameters of the gene expression model in their study were obtained from FiT‐DB (https://fitdb.dna.affrc.go.jp/).

### 2.6 Detection of fungal and viral infection of rice leaves using de novo assembly of unmapped reads to the rice reference transcriptome

Since Lasy‐Seq (Kamitani et al., 2019) detects RNA with poly(A) tails, unmapped reads of the rice reference genome can contain RNA of fungi and viruses with poly(A) tails. To clarify whether the rice plants sampled in this study were infected by fungi or viruses, we conducted de novo assembly of unmapped reads to the rice reference genome (Supporting Information: Figure S9). Raw sequence reads were merged based on the experiments and conditions of each sample. Reads were then trimmed using Trimmomatic version 0.33 (Bolger et al., 2014) with the parameters described above and were then mapped to the rice reference genome using Bowtie2 with default parameters, except setting *N *= 1. After extracting the unmapped reads and removing the duplicated reads, de novo assembly was conducted using Trinity with default parameters. After removing redundant reads using CD‐HIT (Fu et al., 2012), 19 contigs were identified. Each contig was annotated using BLASTn for nucleotides (Camacho et al., 2009) (Supporting Information: Figure S9, Supporting Information: Table S3).

### 2.7 Statistical analysis

All statistical analyses were performed using R software version 3.5.3 (R core Team, 2019). Specifically, differentially expressed gene (DEG) analysis was conducted using R package TCC version 1.20.0 (Sun et al., 2013; Tang et al., 2015). Normalization was conducted using iDEGES/edgeR (Robinson et al., 2010) with a false discovery rate (FDR) of 0.1, and DEG detection was conducted using edgeR with FDR = 0.05. Gene enrichment tests for GO and Kyoto Encyclopaedia of Genes and Genomes (KEGG) (Kanehisa & Goto, 2000) pathways were conducted using the R package GO.db version 3.6.0 (Carlson, 2018) and KEGG.db version 3.2.3 (Carlson, 2016), respectively, as described by Nagano et al. (2019). The FDR was controlled using Benjamini and Hochberg's method (Benjamini & Hochberg, 1995) with FDR = 0.05. Log_2_ (rpm) was calculated as log_2_(rpm + 1). Multiple comparison tests for starch and sucrose contents were conducted using the R package car version 3.0.10.

## 3 RESULTS

### 3.1 Reproduction of environmental field conditions with SmartGC

SmartGC can control irradiance with a 1‐second resolution and temperature and relative humidity with a 1‐minute resolution (Supporting Information: Figure S1a–d), enabling the reproduction of fluctuating field environments. We grew rice plants in SmartGC for 17 days under square‐wave conditions. Subsequently, we transferred the plants to five different treatment conditions, where they were left to acclimate for 2 days. The leaves were sampled on the third day (Figure 1a). First, we conducted experiments in the field (FIELD) to measure irradiance (light, L), temperature (T) and relative humidity (H) (Supporting Information: Figure S1e,f). We then conducted an experiment in SmartGC simulating the environmental factors of FIELD (fluctuating L, T and H; FL/FTH). A square‐wave condition experiment was also conducted using SmartGC (constant L, T and H; CL/CTH). To distinguish the effect of environmental factors on rice transcriptomes, we also set conditions where only irradiance (FL/CTH) or temperature and humidity (CL/FTH) were fluctuating, while the other factors were held constant. SmartGC successfully simulated irradiance, temperature and humidity fluctuations for FIELD, except for simulations with high irradiance, a low R:FR ratio, and high or low humidity due to limitations in the setting of the growth chamber (Figure 1b,c and Supporting Information: Figures S2,S4 and S5).

### 3.2 Evaluation of transcriptome similarity between field and controlled environments

We sampled rice leaves from four biological replicates in each condition every 2 h for 24 h (260 total samples) and conducted RNA‐Seq analysis (Experiment_1; Figure 1a, Supporting Information: Figures S4–S6, Supporting Information: Table S1). Sampling was conducted in the dark from 19:00 to 5:00, and in the light from 7:00 to 17:00. Another experiment was conducted under four controlled conditions to investigate responses around dawn and dusk (Experiment_2; 204 samples obtained from 17 time‐points [5:00 to 19:00] with three biological replicates). In this experiment, sampling was conducted in the dark from 5:00 to 5:30 and from 18:30 to 19:00, and in the light from 6:00 to 17:30. In FIELD, it was after dusk (17:55) at 18:00, but the irradiance (PFD) was above 1 µmol photon m^−2^ s^−1^. Thus, the sampling at 18:00 was conducted in the light in FL/FTH and FL/CTH, and in the dark in CL/CTH and CL/FTH (Supporting Information: Figure S4b). The differences in rice plant growth among the five conditions were not obvious; therefore, leaves at the same stage were sampled in all conditions.

We evaluated transcriptome similarity between conditions using correlation and principal component analysis (PCA). The pairwise Pearson's correlation coefficient (*r*) for each time‐point and condition tended to be higher for FIELD versus FL/FTH than for FIELD versus the other conditions (Figure 1d). The number of genes with *r* > 0.7 and the number of statistically significant genes by correlation (*p*‐value adjusted for multiple comparison test [*q*‐value] < 0.05) were highest for FL/FTH and lowest for CL/CTH (Figure 1e,f). These results indicate that FL/FTH reproduced the rice transcriptome dynamics of FIELD better than CL/CTH. In the Experiment_1 PCA, PC1 separated the samples harvested in the morning from those harvested in the afternoon, and PC2 separated those harvested in the light from those harvested in the dark (Figure 1g and Supporting Information: Figure S10). Consequently, samples were ordered by time, suggesting that the diurnal transcriptome dynamics were similarly influenced by circadian changes under the five conditions. Hierarchical clustering based on Pearson's correlation coefficient separated the samples by sampling time‐points (Figure 1i and Supporting Information: Figure S11a). However, the 9:00 CL/CTH samples were clustered with 11:00 samples of all five conditions, whereas the 9:00 samples of the other conditions were clustered together (Figure 1i and Supporting Information: Figure S11a). Furthermore, 19:00_2 (24 h after the start of sampling at 19:00) CL/CTH and CL/FTH samples were clustered with 17:00 samples of all five conditions, whereas the 19:00_2 samples of the other conditions were clustered together (Figure 1i and Supporting Information: Figure S11). These results suggest that the internal time progression of the samples was faster around 9:00 in CL/CTH and slower around 19:00_2 in CL/CTH and CL/FTH compared with the other conditions, reflecting the differences in irradiance. This was also supported by the internal time inference of transcriptome samples using the molecular timetable method (Higashi et al., 2016; Ueda et al., 2004) (Supporting Information: Figure S8, Supporting Information: Table S2). These results are consistent with that of a previous study on statistical modelling with transcriptome data (Matsuzaki et al., 2015), which showed that the internal time progression in conventional growth chamber conditions was faster after lights‐on and slower before lights‐off than in the field. Experiment_2 samples were also separated by time, excluding those in the morning, using PCA and hierarchical clustering (Figure 1h,j and Supporting Information: Figures S11b and S12). In the Experiment_2 PCA, PC1 separated the samples based on the progression of time, and PC2 separated the samples according to the average irradiance of the sampling time (Figure 1h and Supporting Information: Figure S12). Samples at 7:00 under CL/CTH were clustered with the 8:00 FL/CTH and CL/FTH samples and the 9:00 FL/FTH samples (Figure 1j and Supporting Information: Figure S12). This time lag between conditions continued until 11:00, suggesting that the morning internal time progression was affected by temperature, humidity and irradiance.

**Figure 2:**
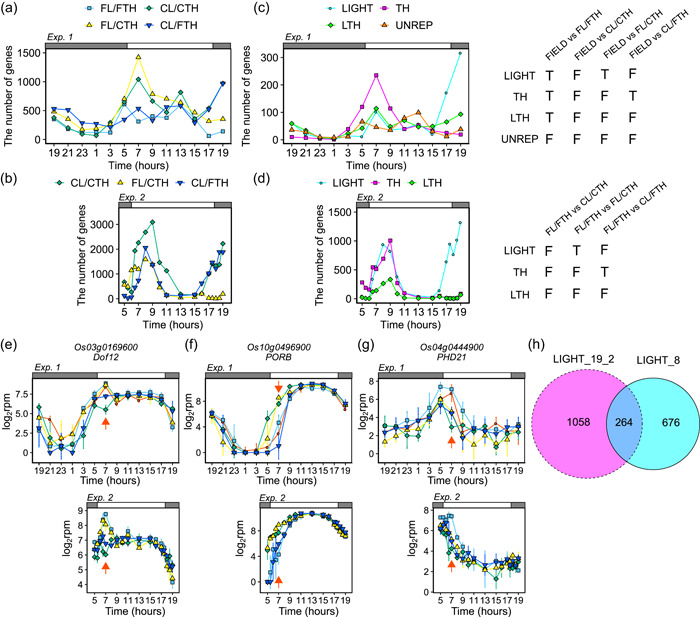
Light affects transcriptome dynamics in the morning and evening while temperature affects them only in the morning. (a) and (b) The number of DEGs between (a) FIELD and the other conditions at each time‐point in Experiment_1 and (b) FL/FTH and the other conditions at each time point in Experiment_2. (c) and (d) The number of genes affected by environmental conditions at each time‐point in (c) Experiment_1 and (d) Experiment_2. Each gene set was selected using the schemes shown in the table. DEGs between conditions that were included and not included in each gene set are shown as T and F, respectively. LIGHT, genes affected by light; TH, genes affected by air temperature; LTH, genes affected by light and air temperature; UNREP, genes whose expression was regulated by factor(s) other than light and air temperature. (e)–(g) Expression of (e) LIGHT, (f) TH and (g) LTH genes at 7:00 in Experiment_1 and Experiment_2. Points indicate means, and error bars indicate standard deviations (*n* = 4 and *n* = 3 in Experiment_1 and Experiment_2, respectively). Red arrows indicate 7:00. rpm, reads per million. (h) Venn diagram of LIGHT genes in the morning (8:00) and evening (19:00_2).

We then evaluated transcriptome similarity between conditions using DEG analysis at each sampling time‐point. We compared FIELD with the other conditions using DEG analysis in Experiment_1 (Supporting Information: Table S4). DEG analysis indicates the DEGs between FIELD and the other conditions. There tended to be fewer DEGs in FL/FTH than in other conditions (Figure 2a). This is consistent with the results that the rice transcriptome dynamics in FIELD were better reproduced under FL/FTH than in the other conditions (Figure 1d–f,i). The number of DEGs peaked at 7:00 in CL/CTH and FL/CTH, and at 19:00_2 in CL/CTH and CL/FTH (Figure 2a). Since temperature and humidity were equal in CL/CTH and FL/CTH, these results suggest that the difference between FIELD and CL/CTH in the morning was mainly due to temperature and/or humidity. In contrast, irradiance was equal for CL/CTH and CL/FTH, suggesting that the differences between FIELD and CL/CTH in the evening were mainly due to irradiance. Unlike at 19:00_2, no clear differences between FIELD and CL/CTH were observed at 19:00 (Figure 2a). This may reflect the weather differences before sampling: the second day was cloudy, while the third day was sunny (Figure 1b,c). We investigated the overlap of DEGs at each time‐point and characterized genes affected by environmental conditions into four types (Figure 2c,e–g): genes affected by light (LIGHT); genes affected by temperature and humidity (TH); genes affected by light, temperature and humidity (LTH); and genes whose expression in the field was not reproduced under controlled conditions (UNREP). The number of UNREP genes peaked at 13:00 (Figure 2c). This might reflect the high irradiance in FIELD, which was not simulated by SmartGC (Figure 1b,c). The number of TH genes tended to be higher in the morning, peaking at 7:00, while the number of LIGHT genes tended to be higher in the evening, peaking at 19:00_2 (Figure 2a). Additionally, the number of LTH genes was highest at 7:00 and second highest at 19:00_2 (Figure 2c), suggesting the importance of morning light conditions.

The number of DEGs between FL/FTH and the other conditions in Experiment_2 peaked in the morning and evening for CL/CTH and CL/FTH, and only peaked in the morning for FL/CTH (Figure 2b, Supporting Information: Table S5). The overlap of DEGs in Experiment_2 showed that the number of LIGHT genes was high in the morning and evening, while that of TH genes was high only in the evening (Figure 2d). These results confirm the findings from PCA and hierarchical clustering (Figure 1g–j). Interestingly, >50% of the LIGHT genes at 8:00 and 19:00_2 overlapped (Figure 2h), suggesting that the effect of irradiance on transcriptome dynamics was different between the morning and evening. The number of TH genes was higher than that of LTH and LIGHT genes from 5:00 to 6:00 (Figure 2d), indicating that temperature and humidity began affecting the transcriptome before dawn. In contrast, the number of LTH and LIGHT genes increased from 6:00 to 6:30, indicating that light began affecting the transcriptome 0.5–1 h after dawn. Since the start of dawn only differed by 10 min between FL/FTH and CL/CTH (Supporting Information: Figure S4), the gradual versus sudden increase of irradiance, and not the difference in the timing of dawn, caused the upregulation of LTH and LIGHT genes after dawn. This is consistent with previous studies on Arabidopsis, and it may have been caused by the regulation of gene expression by phytochrome A after dawn (Seaton et al., 2018). Likewise, the number of TH genes increased from 1 h before dusk (16:00–17:00) (Figure 2d), indicating that a gradual versus sudden decrease of irradiance, and not the difference in the timing of dusk, caused the increase of TH genes before dusk. Overall, these results suggest that gradual versus sudden changes in irradiance affect transcriptome dynamics in the morning and evening, whereas changes in temperature and/or humidity only affect transcriptome dynamics in the morning. The number of DEGs between FL/FTH and the other conditions in Experiment_2 tended to be higher than that between FIELD and the other conditions in Experiment_1 (Figure 2a–d). This may have resulted from fewer environmental factors affecting transcriptome in SmartGC than in the field.

Although we could not distinguish the effects of temperature and humidity on the transcriptome, the effect of temperature could be greater than that of humidity (Nagano et al., 2012). Therefore, we considered the effect of temperature or humidity as the effect of temperature in subsequent discussions.

### 3.3 Circadian clock genes respond to fluctuating irradiance and temperature

**Figure 3:**
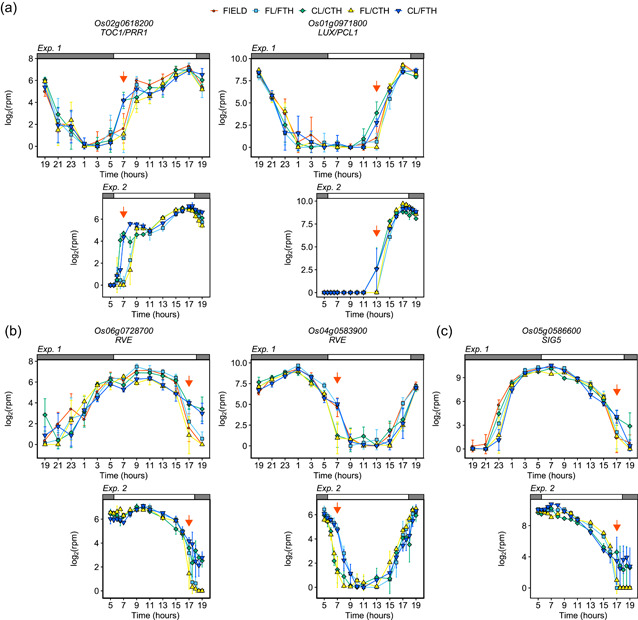
Circadian clocks under field and controlled conditions. Expression of (a) *TIMING OF CAB EXPRESSION 1*/*PSEUDO‐RESPONSE REGULATOR 1* (*TOC1*/*PRR1*), *LUX ARRHYTHMO*/*PHYTOCLOCK 1* (*LUX*/*PCL1*), (b) genes encoding *REVEILLE*, and (c) genes encoding sigma factor *SIG5* in Experiment_1 and Experiment_2. Points indicate means, and error bars indicate standard deviations (*n* = 4 and *n* = 3 in Experiment_1 and Experiment_2, respectively). Red arrows indicate the sampling times discussed in the manuscript. rpm, reads per million.

Among the circadian clock genes (Figure 3 and Supporting Information: Figures S13–S15), *TIMING OF CAB EXPRESSION 1*/*PSEUDO‐RESPONSE REGULATOR 1* (*TOC1*/*PRR1*) and *LUX ARRHYTHMO*/*PHYTOCLOCK 1* (*LUX*/*PCL1*) expression clearly differed between conditions (Figure 3a). *TOC1/PRR1* expression increased from 7:00 to 9:00 in FIELD, FL/FTH, and FL/CTH, and from 5:00 to 7:00 in CL/CTH and CL/FTH. *LUX*/*PCL1* expression increased from 13:00 to 15:00 in FIELD, FL/FTH, and FL/CTH, and from 11:00 to 13:00 in CL/CTH and CL/FTH. These results suggest that the increases in *TOC1*/*PRR1* expression in the morning and in *LUX*/*PCL1* expression in the daytime were affected by irradiance. In Arabidopsis, *REVEILLE* (*RVE*) genes are positive regulators of *PRR5*, *TOC1*/*PRR1*, and evening complex genes (*ELF3*, *ELF4*, and *LUX/PCL1*) (Grey et al., 2017). The expression of two *RVE* genes (*Os06g0728700* and *Os02g0680700*) decreased from 15:00 to 17:00 in FIELD, FL/FTH, and FL/CTH, and from 17:00 to 19:00_2 in CL/CTH and CL/FTH, suggesting the involvement of irradiance in their regulation (Figure 3b and Supporting Information: Figure S15). In contrast, expression of the other two *RVE* genes (*Os04g0538900* and *Os02g0685200*) decreased from 7:00 to 9:00 in FIELD, FL/FTH, and CL/FTH, and from 5:00 to 7:00 in CL/CTH and FL/FTH (Figure 3b and Supporting Information: Figure S15), suggesting that their expression was affected by diurnal temperature changes. Nuclear‐encoded sigma factor *SIG5* regulates the expression of some plastid‐encoded genes (Noordally et al., 2013). *SIG*5 expression was lower in FIELD, FL/FTH and FL/CTH than in CL/CTH and CL/FTH at 17:00 and 19:00_2 (Figure 3c). This may indicate that plastid‐encoded gene expression responds to changes in diurnal irradiance via *SIG5*. Because mRNA was reverse‐transcribed using oligo‐dT primers in our RNA‐Seq method (Kamitani et al., 2019) and plastid‐encoded genes do not have poly‐A tails in their mRNA, we were unable to obtain reliable data on the expression levels of plastid‐encoded genes from our RNA‐Seq analysis. Therefore, further analysis of plastid‐encoded gene expression is necessary. Overall, these results identified circadian clock genes that respond to fluctuating irradiance and temperature. Further studies are needed to clarify the role of individual genes in response to fluctuating environmental conditions.

### 3.4 Diurnal fluctuation of irradiance affects rice leaf sugar metabolism

**Figure 4:**
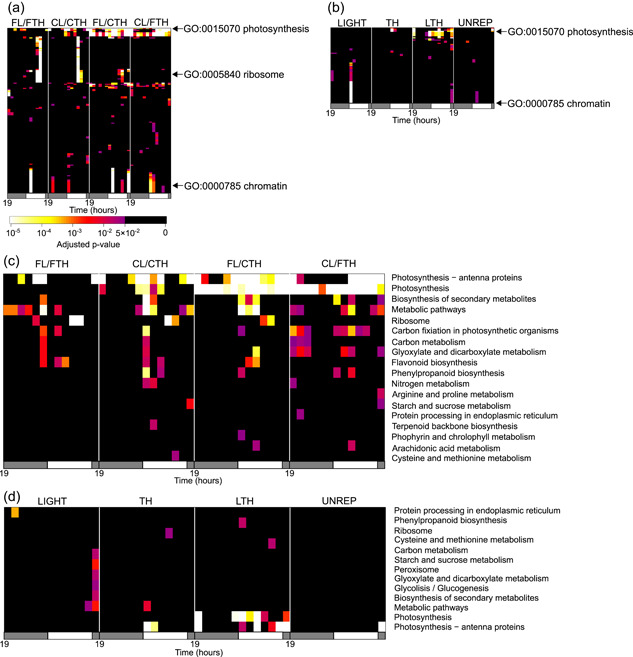
Identification of gene sets affected by fluctuating environmental conditions. Heatmaps of *p*‐values (Fisher's exact test, two‐sided) for significant genes with (a) and (b) a particular gene ontology (GO) and (c) and (d) a particular KEGG pathway (row) at each time and condition (column) in (a) and (c) DEGs between FIELD and the other conditions and (b) and (d) LIGHT, TH, LTH and UNREP genes in Experiment_1. GO and KEGG pathways that have at least one significant (adjusted *p* < 0.05) time‐point and condition are shown in the heatmaps.

To characterize the environmentally‐affected genes, we tested for enrichment of genes with annotations in the DEGs detected above (Figure 4 and Supporting Information: Figure S16, Supporting Information: Tables S6–S13). A total of 7564 and 2942 genes, which have at least one gene ontology (GO) annotation or which belong to one KEGG pathway, respectively, were used for the enrichment test. Genes annotated for photosynthesis (GO:0015979; KEGG pathway: dosa00195) were significantly enriched in DEGs and LTH genes in Experiment_1 (Figure 4), suggesting that fluctuations in irradiance and temperature affected photosynthesis‐related gene expression. Genes annotated for photosynthesis and photosynthesis‐antenna proteins (KEGG pathway: dosa00196) or photosynthetic light harvesting (GO:0009765) were significantly enriched in TH genes at 7:00–9:00 in Experiment_1 and 5:00–6:30 in Experiment_2 (Figure 4b,d and Supporting Information: Figure S16b,d). The expression of some photosynthetic light‐harvesting genes increased before dusk, and this increase occurred earlier for CL/CTH and FL/CTH than for FIELD, FL/FTH and CL/FTH (Supporting Information: Figure S17). These results indicate that photosynthetic light harvesting‐related genes are examples of genes whose expression in the morning is affected by gradual changes and fluctuations in temperature. This is consistent with the gene expression model suggested by Nagano et al. (2012). Among the 15 genes annotated for photosynthesis‐antenna proteins or photosynthetic light harvesting, the expression of nine genes was affected by night temperature (Supporting Information: Figure S17, Supporting Information: Table S14).

**Figure 5:**
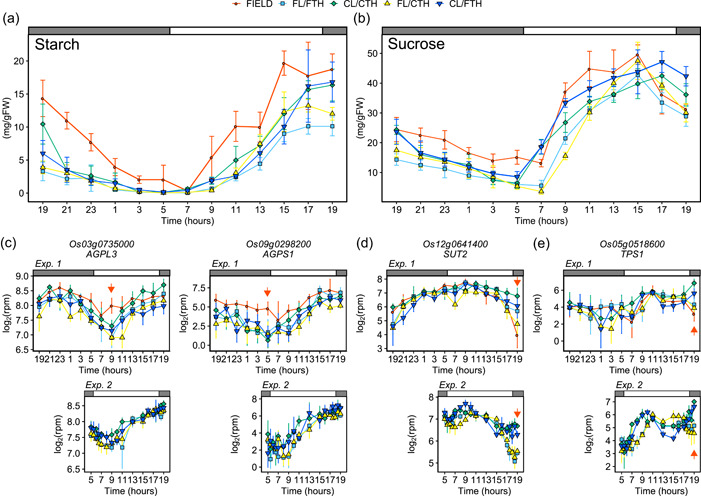
Coordination of leaf sugar metabolism and diurnal change in irradiance. (a) and (b), Diurnal change in (a) starch and (b) sucrose content in leaves of each condition in Experiment_1. Results of multiple comparison analyses between conditions using the Tukey–Kramer method are shown in Supporting Information: Table S15. (c)–(e) Expression of genes encoding (c) large and small subunits of adenosine diphosphate‐glucose pyrophosphorylase (*AGPL3* and *AGPS1*, respectively), (d) tonoplast‐localized sucrose transporter (*SUT2*), and (e) trehalose phosphate synthase (*TPS1*) in Experiment_1 and Experiment_2. Points indicate means, and error bars indicate standard deviations (*n* = 4 and *n* = 3 in Experiment_1 and Experiment_2, respectively). Red arrows indicate the sampling times discussed in the manuscript. FW, fresh weight; rpm, reads per million.

Significant enrichment of sugar metabolism genes was observed in the evening. Genes annotated for starch and sucrose metabolism (KEGG pathway: dosa00500) were significantly enriched in DEGs in FIELD versus CL/CTH and LIGHT at 19:00_2 in Experiment_1, and in DEGs in FL/FTH versus CL/CTH from 17:00 to 19:00, FL/FTH versus CL/FTH at 17:30–19:00, and LIGHT genes at 17:30–19:00 in Experiment_2 (Figure 4c,d and Supporting Information: Figure S16c,d). These are examples of genes whose evening expression is affected by fluctuations in irradiance. In Arabidopsis, differences in irradiance between sinusoidal and square‐wave conditions affect diurnal changes in carbohydrate content (Annunziata et al., 2017, 2018). To clarify the effect of environmental conditions on rice leaf carbohydrate metabolism, we measured the starch and sugar contents in Experiment_1 leaves. Carbohydrate content, especially of sucrose, reflected the differences in diurnal changes of irradiance between conditions (Figures 1b,c and 5a,b, Supporting Information: Table S15). In CL/CTH and CL/FTH, starch and sucrose content decreased from dusk to dawn and then increased from dawn to dusk. For sucrose content, a delayed increase at dawn and an early decrease before dusk were observed in FIELD, FL/FTH and FL/CTH, which is consistent with previous results on Arabidopsis (Annunziata et al., 2017, 2018). Delayed increase in Arabidopsis leaf starch at dawn was also observed under sinusoidal conditions with irradiance, but this trend was less prominent in rice. This reflects leaf carbohydrate composition; rice mainly stores sucrose, whereas Arabidopsis mainly stores starch (Okamura et al., 2017). Although the diurnal trends of changes in starch and sucrose content were similar between FIELD, FL/FTH and CL/FTH, starch and sucrose content tended to be higher in FIELD than in FL/FTH and FL/CTH. This may have been due to the inability of SmartGC to simulate high irradiance during sunny conditions at daytime (Figure 1b,c) or to the differences in the light source (sunlight in FIELD vs. LED in SmartGC).

Accordingly, with the differences in leaf carbohydrate content, sugar metabolism genes clearly differed between the conditions. Expression of *AGPL3* and *AGPS1*, which encode adenosine diphosphate‐glucose pyrophosphorylase (AGP), a key enzyme in starch synthesis (Okamura et al., 2017), tended to be higher in FIELD than in the other conditions (Figure 5c). Evening expression of other starch synthesis genes, such as starch synthases (*SSI*, *SSIIb* and *SSIIIb*) and granule‐bound starch synthase (*GBSSII*), exhibited similar trends in the changes to irradiance; expression decreased earlier in FIELD, FL/FTH and FL/CTH than in CL/CTH and CL/FTH (Supporting Information: Figure S18a). This trend was also observed for sugar metabolism and signalling‐related gene expression (Figure 5d,e, Supporting Information: Figures S18b and S19). For example, expression of sugar transporter (*SUT2*), which regulates carbon export from source leaves to sink organs in rice (Eom et al., 2011), exhibited a similar trend (Figure 5d). Furthermore, genes encoding trehalose 6‐phosphate synthase and trehalose 6‐phosphate phosphatase, which belong to the trehalose biosynthesis pathway and play a significant role in sugar signalling (Figueroa & Lunn, 2016; Paul et al., 2018), exhibited similar trends in the leaf sucrose content (Figure 5e, Supporting Information: Figure S19). Overall, these results indicate that differences in diurnal changes in irradiance between FIELD and CL/CTH conditions affected the carbon status and expression of sugar metabolism genes in rice leaves, especially in the evening.

### 3.5 Field‐specific expression of genes related to biotic and abiotic stress

**Figure 6:**
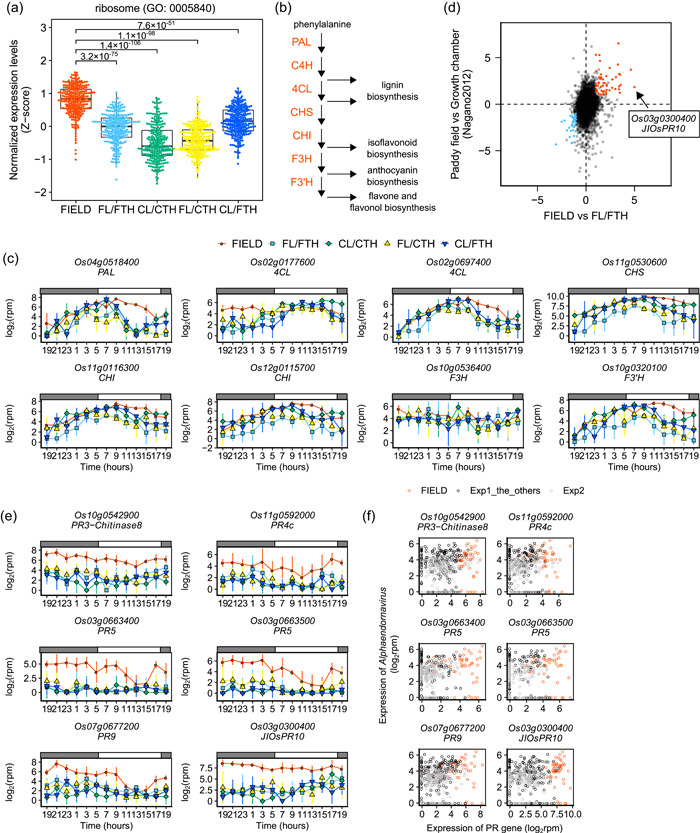
Field‐specific expression of genes related to biotic and abiotic stress. (a) Expression of ribosome‐related genes is upregulated in FIELD. Boxplot showing the normalized expression levels (*z*‐score) of genes with annotations for ribosomes (GO:0005840) between FIELD and the other conditions at 13:00 in Experiment 1. Adjusted *p*‐values of Wilcoxon rank‐sum test between FIELD and the other conditions are shown. (b) Outline of the phenylpropanoid biosynthesis pathway. Enzymes catalysing each reaction are shown in red. CHI, chalcone isomerase; CHS, chalcone synthase; C4H, cinnamate 4‐hydroxylase; F3H, flavanone 3‐hydroxylase; F3'H, flavonoid 3‐hydroxylase; 4CL, 4‐coumarate:coenzyme A ligase; PAL, l‐phenylalanine ammonia‐lyase. (c) Expression of genes related to the phenylpropanoid biosynthesis pathway. Points indicate means, and error bars indicate standard deviations (*n* = 4). (d) Scatter plot showing the differences in the mean expression value between FIELD and FL/FTH in this study and between paddy field and the growth chamber in Nagano et al. (2012). Genes whose mean value of expression was more than 2.0 times higher or lower in both experiments and significantly different between FIELD and FL/FTH at one or more time points are shown as red and blue points, respectively. (e) PR genes whose expression was upregulated in FIELD. Points indicate means, and error bars indicate standard deviations (*n* = 4). (f) Scatter plot showing the relationship between the expression of *Alphaendornavirus* (Moriyama et al., 1995) and PR genes, which was upregulated in FIELD.

Gene enrichment tests for DEGs showed gene expression specific to FIELD. Genes annotated for ribosomes (GO:0005840; KEGG pathway: dosa03010) were significantly enriched in DEGs in FIELD versus FL/FTH, CL/CTH and FL/CTH at 13:00 and 15:00 (Figure 4a). Ribosome‐annotated gene expression was significantly higher in FIELD than in the other conditions (Figure 6a). These results suggest that ribosome‐related gene expression was upregulated by unevaluated environmental factor(s) that differed between field and SmartGC experiments. Considering that ribosomal gene expression responds to various biotic and abiotic stresses (Moin et al., 2016), the upregulation of ribosome‐related genes was probably a response to stressors specific to the field environment. Genes annotated for chromatin (GO:0000785) were significantly enriched in DEGs between FIELD and the other conditions, especially in the morning, indicating the difference in chromatin state dynamics induced by sunlight and LEDs (Figure 4a,b and Supporting Information: Figure S20).

Genes annotated for secondary metabolites (KEGG pathway: dosa01110), phenylpropanoid biosynthesis (KEGG pathway: dosa00940), and flavonoid biosynthesis (KEGG pathway: dosa00941) (which were significantly enriched in DEGs in FIELD compared to other conditions), and LTH and UNREP genes (Figure 4c,d). Phenylpropanoid biosynthesis‐related gene expression (Figure 6b) was significantly upregulated in FIELD compared to the other conditions, mainly from 9:00 to 15:00, except for a gene encoding 4‐coumarate:coenzyme A ligase (*Os02g0177600*), which was upregulated at night (Figure 6c). Since phenylpropanoid biosynthesis‐related gene expression is induced by various biotic and abiotic stresses (Dixon & Paiva, 1995; Vogt, 2010), these results suggest that the upregulation of these genes was a response to field environment stresses.

To investigate field‐specific gene expression, we focused on the transcriptome differences between FIELD and FL/FTH. We calculated the mean value of each gene's expression at all time points and extracted genes whose expression was 2× higher or lower in FIELD than in FL/FTH. We also extracted genes whose expression significantly differed between FIELD and FL/FTH at one or more time points. A total of 159 and 78 genes were identified as upregulated and downregulated, respectively, in FIELD (Figure 6d, Supporting Information: Tables S16 and S17). Phenylpropanoid biosynthesis‐related genes were observed among the highly expressed genes in FIELD (Supporting Information: Table S16). We also found several genes encoding pathogenesis‐related (PR) proteins (Figure 6e, Supporting Information: Table S16), which are induced by pathogen attack and are a key component of systemic acquired resistance (SAR) (Backer et al., 2019). *NONEXPRESSOR OF PATHOGENESIS‐RELATED GENES 1* (*NPR1*) is important for establishing SAR and indirectly activating PR gene expression (Backer et al., 2019). However, *NPR1* and the other NPR genes were not upregulated in FIELD (Supporting Information: Figure S21), suggesting that PR gene upregulation was independent of NPR genes. Terpene synthesis‐related genes, which defend against herbivore‐ and pathogen‐caused tissue damage (Yoshitomi et al., 2016), were also upregulated in FIELD (Supporting Information: Figure S22, Supporting Information: Table S17). However, we did not observe any signs of herbivory or herbivorous insects. In addition, herbivory‐induced early defence signalling genes (Ye et al., 2019) were neither upregulated nor downregulated, except for *WRKY30* downregulation in FIELD (Supporting Information: Figure S23). Therefore, PR and terpene synthesis‐related gene upregulation was likely independent of the effect of insects.

Although no pathogen infection symptoms were observed, upregulation of PR and terpene synthesis‐related genes may have resulted from pathogen infection. Therefore, we attempted to detect viral and fungal infections from RNA‐Seq data using our previously reported pipeline (Kamitani et al., 2016) (Supporting Information: Figure S24) and de novo assembly of unmapped reads to the rice reference genome (Supporting Information: Figure S9). The number of reads of viruses (Moriyama et al., 1995) and fungi with poly(A) tails was not specific for FIELD nor correlated with PR gene expression (Figure 6f and Supporting Information: Figures S24 and S25, Supporting Information: Table S3). Although we cannot exclude the possibility of infection by bacteria or viruses without poly(A) tails, these results suggest that upregulation of PR and terpene synthase genes in FIELD was a response to physical environmental factor(s) specific to the field.

To determine whether field‐specific gene expression is also present in paddy‐field rice, we re‐analysed the microarray data of rice leaves sampled from a paddy field and a growth chamber, which had been previously analysed by Nagano et al. (2012). Observations regarding up‐ or downregulation of genes in FIELD were consistent with the previous study (Nagano et al., 2012) (Figure 6d, Supporting Information: Tables S18 and S19), suggesting that the field‐specific gene expression information we obtained is also applicable to paddy‐field rice.

## 4 DISCUSSION

Although differences between plants grown in the field and controlled environments are well known (Poorter et al., 2016), few studies have explored the underlying molecular mechanisms for these. Here, we established an experimental scheme for using laboratory equipment to evaluate plant responses to fluctuating environments. We revealed diurnal transcriptome dynamics in both environments and their fillable and unfillable gaps. Our results complement those that model plant transcriptome responses in field environments (Matsuzaki et al., 2015; Nagano et al., 2012). Gradual changes in irradiance affected transcriptome dynamics in the morning and evening, whereas temperature changes only had an effect in the morning (Figure 2). Accordingly, our statistical model suggested that the number of genes whose expression was affected by a time‐specific temperature was the lowest from noon to dusk (Nagano et al., 2012). The number of genes whose expression was affected by time‐specific daily irradiance was higher during daytime and the highest around noon. There was no difference in the number of genes expressed in the morning and evening. As the plant circadian clock is dawn‐dominant (Edwards et al., 2010; Flis et al., 2016, 2019), the fact that both morning irradiance and temperature affect transcriptome dynamics is likely due to circadian entrainment by irradiance and temperature. Conversely, this suggests that the effect of evening irradiance is independent of circadian regulation. Accordingly, less than a half of the morning and evening LIGHT genes overlapped (Figure 3f). Since sugar metabolism gene expression corresponded to decreased sucrose content in the evening (Figure 5 and Supporting Information: Figures S16 and S17) and diurnal changes in sugar status affect transcriptome dynamics independent of the circadian clock (Cookson et al., 2016; Flis et al., 2016), the effect of evening irradiance potentially depends on differences in carbon status between conditions. We cannot exclude the possibility that morning light and temperature cause additional effects independent of the circadian clock.

We found that the expression of *RVE* genes responded to gradual changes in irradiance and temperature (Figure 3b and Supporting Information: Figure S15a). Since *TOC1*/*PRR1* and *LUX*/*PCL1* are positively regulated by *RVE* in Arabidopsis (Grey et al., 2017) and the expression of two *RVE* genes (*Os06g0728700* and *Os02g0680700*), *TOC1*/*PRR1*, and *LUX*/*PCL1* were affected by a gradual change in irradiance (Figure 3a), the two *RVE* genes might play a role in the regulation of *TOC1/PRR1* and *LUX/PCL1* in rice. The expression pattern suggests that *Os06g0728700* might serve as an activator of *TOC1/PRR1*. This is consistent with a previous study suggesting that the role of *RVE* genes as *TOC1*/*PRR1* activators is conserved in rice (Toda et al., 2019). In contrast, *Os02g0680700* might function as a repressor of *LUX*/*PCL1* rather than an activator (Figure 3b). This would suggest that the positive regulation of *LUX/PCL1* by *RVE* is not conserved in rice. A previous study suggested that *RVE* responded to low carbon status in Arabidopsis (Moraes et al., 2019). Thus, the expression of the two *RVE* genes might be affected by differences in carbon status derived from either gradual or sudden changes in irradiance. The expression patterns of the other two *RVE* genes (*Os04g0538900* and *Os02g0685200*) were affected by temperature (Figure 3b and Supporting Information: Figure S15a). However, the expression of core circadian clock genes including *TOC1/PRR1* did not show clear difference between different temperature conditions (Figure 3a and Supporting Information: Figure S13). As some *RVE* genes may be involved in the circadian clock output pathway of Arabidopsis (Rawat et al., 2009; Zhang et al., 2007), the impact of the temperature‐affected *RVE* genes on circadian regulation might be different from that of the *RVE* genes affected by irradiance. Further studies are needed to clarify the role of *RVE* in circadian oscillator regulation through responses of rice to fluctuating environmental stimuli.

Field plants experience various biotic and abiotic stresses, such as insect and pathogen attack, wind and UV light, which were not simulated by SmartGC. In FIELD conditions, we found upregulated genes related to ribosomes, phenylpropanoid biosynthesis and pathogen defence (Figure 6), all of which are responses to biotic and abiotic stress (Ali et al., 2018; Dixon & Paiva, 1995; Moin et al., 2016; Vogt, 2010). This indicates that plants cope with the field environment by upregulating stress‐responsive genes, despite the less stressful field environment compared to the stress‐treatment experiments. Alongside the finding that these genes are also upregulated in paddy fields (Figure 6f), our study suggests that these genes can be targets for rice productivity improvement in the field.

In addition to the presence or absence of UV light, factors related to light quality that were not simulated by SmartGC (Supporting Information: Figure S2), such as the R:FR ratio and the proportion of blue light, may have caused the differences between the plants grown in the field and with SmartGC. In this study, the R:FR ratio in SmartGC was higher and more fluctuating than that in FIELD (Supporting Information: Figure S2). This may have affected phytochrome signalling and phytochrome‐dependent processes such as the circadian clock and leaf development (Soy et al., 2016). In Arabidopsis, a higher R:FR ratio between field and controlled environments has been shown to cause differences in the expression patterns of FT genes, and these differences involved phytochrome A (Song et al., 2018). Inactivation of phytochrome B by far‐red light at the end of the day affects leaf development (Romanowski et al., 2021) and phytochrome B also acts as a temperature sensor (Jung et al., 2016; Legris et al., 2016). Therefore, a high R:FR ratio could affect transcriptome dynamics in a wide range of biological processes. Furthermore, the proportion of blue light also affects transcriptome dynamics (Pedmale et al., 2016). Since the proportion of blue light affects phenylpropanoid synthesis (Huché‐Thélier et al., 2016), it is possible that higher expression levels of genes annotated for phenylpropanoid biosynthesis result from differences in the proportion of blue light, as well as other field‐specific environmental factors (Figure 6). It is necessary to further explore the effects of the R:FR ratio and the proportion of blue light on transcriptome dynamics in the future.

The differences between sunlight in the field and LED light in controlled conditions caused differences in the transcriptome and sugar metabolism between the treatment groups. The starch and sucrose contents in the leaves were higher in FIELD than in the other conditions (Figure 5a,b). One reason for this is the inability of SmartGC to simulate the high irradiance that occurs during daytime (Figure 1b,c). It is also possible that the light source affected the irradiance received by the whole plant. Since the light source of SmartGC was located above the rice plants (Supporting Information: Figure S1), the irradiance received on the side of the rice plants was much less than that received at the top of the plants. In contrast, rice plants grown in the field received irradiance on the sides as well as the top because sunlight includes both diffused and direct light. Furthermore, the irradiance from LED light received by plants in SmartGC decreases with increasing distance from the light source, while that from sunlight does not (Niinemets & Keenan, 2012; Poorter et al., 2012). Furthermore, diffuse light reaches the lower part of the plant canopy more efficiently than does direct light (Li et al., 2014). Collectively, these factors mean that the total irradiance received by the plants in SmartGC is less than that received in the field. Considering that the carbohydrate content in CL/CTH and CL/FTH was lower than that in FIELD (Figure 5a,b), the differences in irradiance received by the whole plant likely had a greater effect on carbohydrates than the differences in irradiance during daytime. This might also explain the differences between FIELD and FL/FTH at midday. The number of UNREP genes peaked at 13:00 in Experiment_1 (Figure 2c). Because the photoreceptors were completely saturated by the irradiance at this time of the day, this result might not be related to light signalling. However, the difference in irradiance received by the whole plant between FIELD and FL/FTH might explain the differences observed in the transcriptome, as these may have occurred due to differences in carbon metabolism. As GO terms and KEGG pathways were not significantly enriched in UNREP genes at 13:00 (Figure 4b,d), further studies are required to clarify the mechanisms underlying the differences between FIELD and FL/FTH at midday.

Although the starch and sucrose contents were higher in FIELD than in the other conditions, their trends in FIELD were simulated in FL/FTH (Figure 5a,b). These trends were consistent with previous results on Arabidopsis (Annunziata et al., 2017). In Arabidopsis, starch accumulation in leaves during the day depends on the environmental conditions and the length of the day (Moraes et al., 2019; Stitt & Zeeman, 2012). Similarly, the starch and sucrose content in rice leaves is affected by the length of the day (Okamura et al., 2017). Future studies should thus examine the effects of fluctuating irradiance on starch and sugar metabolism under different photoperiods.

It is possible that the preculture environmental conditions and the acclimation duration affected the transcriptome dynamics in this study. The photoperiods were shortened from 14 h in the preculture to 12 h and 11–37 min in each growth condition (Figure 1a). The effect of shortening the photoperiod was buffered in our experimental scheme because we grew plants for 2 days before sampling to acclimate the rice plants to each condition. However, we cannot rule out the possibility that shortening the photoperiod may have affected the transcriptomes. Extending the acclimation period might better buffer the effects of preculture environmental conditions on the transcriptomes. The evaluation of the effect of these factors on transcriptomes will contribute to the improvement of the experimental scheme established in this study.

We demonstrated the utility of SmartGC for understanding plant responses to fluctuating environments; however, SmartGC cannot completely reproduce field environments, and unfillable gaps remain between plants grown in the field and those grown in SmartGC. To overcome these gaps, it may be practical to control the light quality by increasing the output of far‐red light to decrease the R:FR ratio in SmartGC, and by also including UV‐A and UV‐B light. Furthermore, it would be beneficial to clarify simpler conditions than those used in this study to simulate field environments, with the goal of mimicking field‐grown plants more easily and cost‐effectively than SmartGC. Our results suggest that it is important to include gradual changes in the environmental factors of irradiance and temperature to accurately mimic the conditions of plants grown in the field, although this may depend on the target organs or the objectives of the experiment. However, to clarify the most critical factors for simulating plants grown in the field, we need to further evaluate plant responses to environmental factors such as humidity, light quality, and light fluctuation, which are important yet thus far insufficiently examined features of the field environment. It will also be important to determine the extent to which conditions can be appropriately simplified by quantifying the effects of simplification on plants using SmartGC and transcriptome analysis.

SmartGC is especially useful for difficult‐to‐conduct field experiments, such as those using genetically modified plants, radioisotopes, or rare environmental conditions. Moreover, SmartGC can be used to predict the effects of future climate change on plants by allowing the evaluation of plants grown in a simulated environment. In addition, SmartGC can contribute to improvements in modelling plant transcriptome dynamics. Since transcriptome models can be used to predict the environmental responses of plants, much effort has been made to improve the models. For example, Urquiza‐García and Millar (2021) introduced absolute units of transcription (Flis et al., 2015) to the mathematical model of the circadian clock. Models predicting plant transcriptomes in the field from training data obtained from controlled environments will be improved by using data obtained from the simulated field environment of SmartGC because the plant transcriptome training data will be more similar to field data than would data obtained from plants grown in a conventional growth chamber. Furthermore, statistical models that use data obtained from paddy fields as training data have low predictive power under environments that rarely occur in the field (Nagano et al., 2012). Since SmartGC can simulate rare environmental conditions in the field, incorporating transcriptome data obtained from plants grown in SmartGC will improve these models. Further studies utilizing SmartGC are needed to bridge the gap between field and laboratory studies and to facilitate a comprehensive understanding of plant responses to field environments.

## Supporting information

Supporting information.

Supporting information.

## Data Availability

The scripts used in this study are available at https://github.com/naganolab/SmartGC_RNA-Seq. All datasets generated and/or used in this study are available in the Sequence Read Archive (SRA) under accession number PRJNA726716.
