# Supplementary material for: Fillable and unfillable gaps in plant transcriptome under field and controlled environments
Source: Plant Cell Environ. 2022 Jun 21;45(8):2410–27. doi: 10.1111/pce.14367 (PMC9544781; doi:10.1111/pce.14367)
Supplement: Supplementary file 1 — Supporting information. [file PCE-45-2410-s001.docx]

**
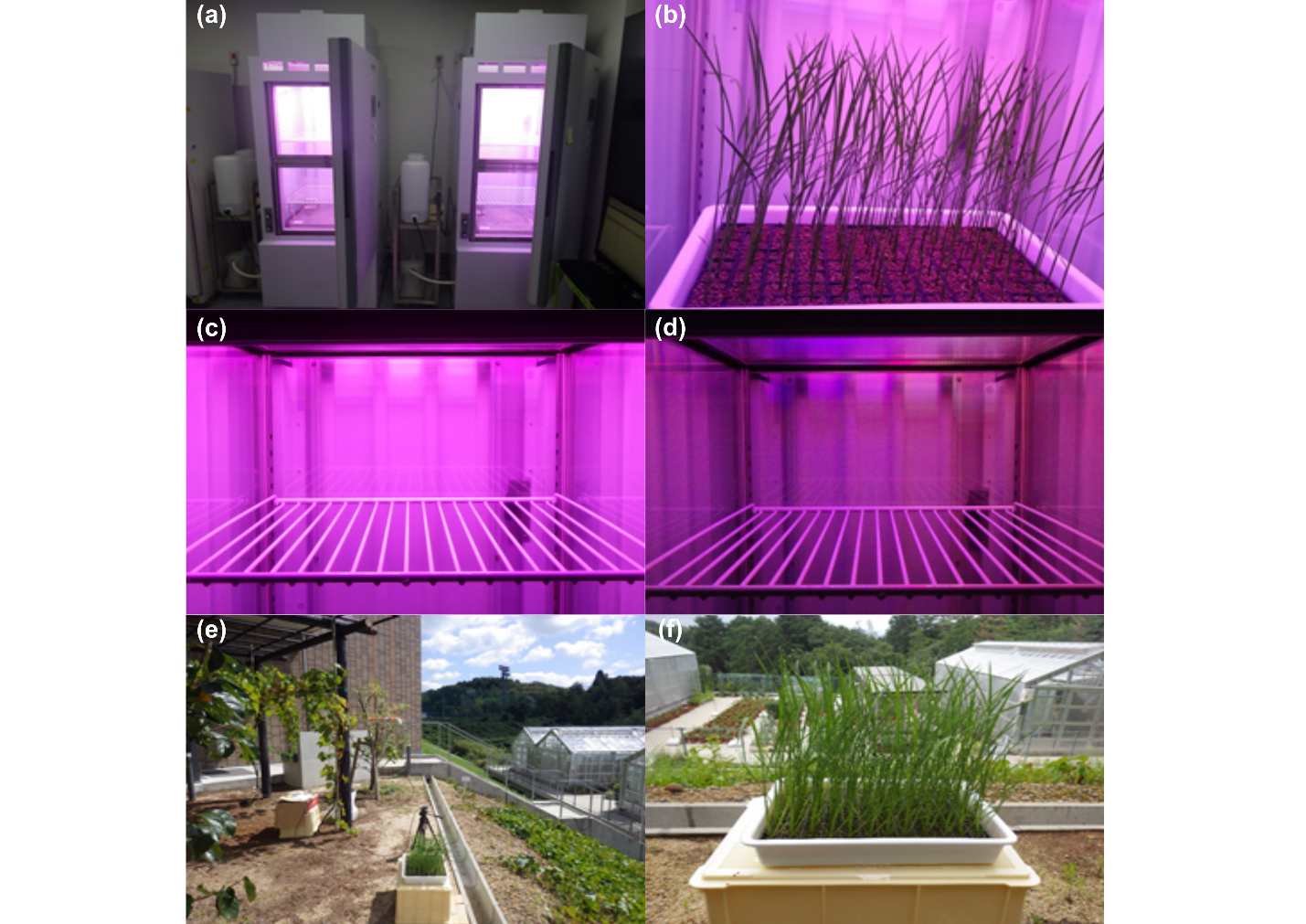
Figure S1 SmartGC system and the field used in this study.** **(a)** The entire SmartGC system. **(b)** Rice grown in SmartGC 14 days after sowing. **(c, d)** The inside of SmartGC. The output value of the light source was **(c)** 1000 sv and **(d)** 15 sv. **(e)** The field used in this study. **(f)** Rice grown in the field 17 days after sowing.

**
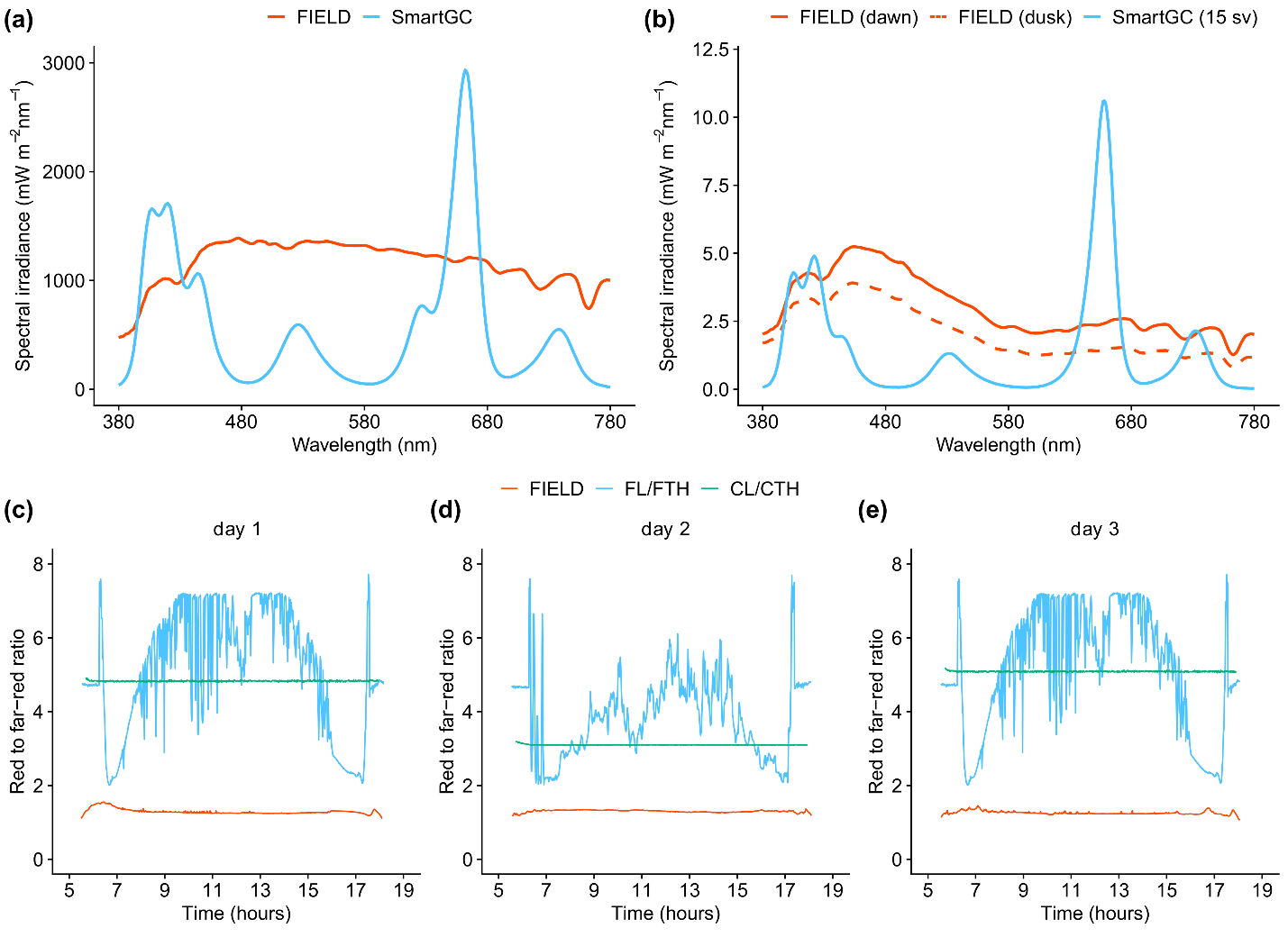
Figure S2** **Spectrums and red to far-red ratio (R:FR) of the irradiance in the field and SmartGC.** **(a, b)** The spectrums of the sunlight and in SmartGC at **(a)** midday and **(b)** dawn and dusk of the sampling day. The output values of the light of the SmartGC were **(a)** 1000 sv and **(b)** 15 sv. **(c**–**e)** Red (655–665 nm) to far-red (730–740 nm) ratio of the irradiance in FIELD, FL/FTH, and CL/CTH conditions on the **(c)** first, **(d)** second, and **(e)** third days.


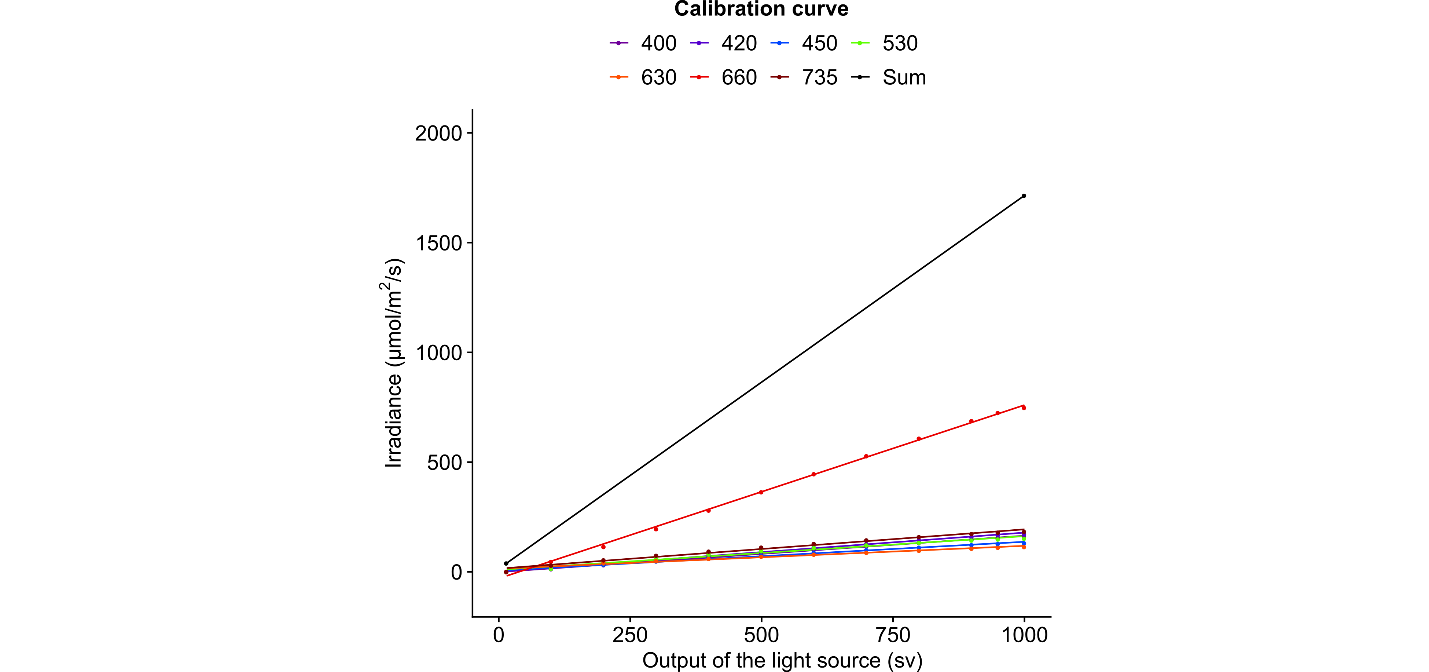


**Figure S3** **Calibration curve of the output of the light source of SmartGC versus irradiance.** Calibration curve for seven types of LED light and the sum of all LEDs are shown. The unit of irradiance is photon flux density (PFD) defined over 380–780 nm.


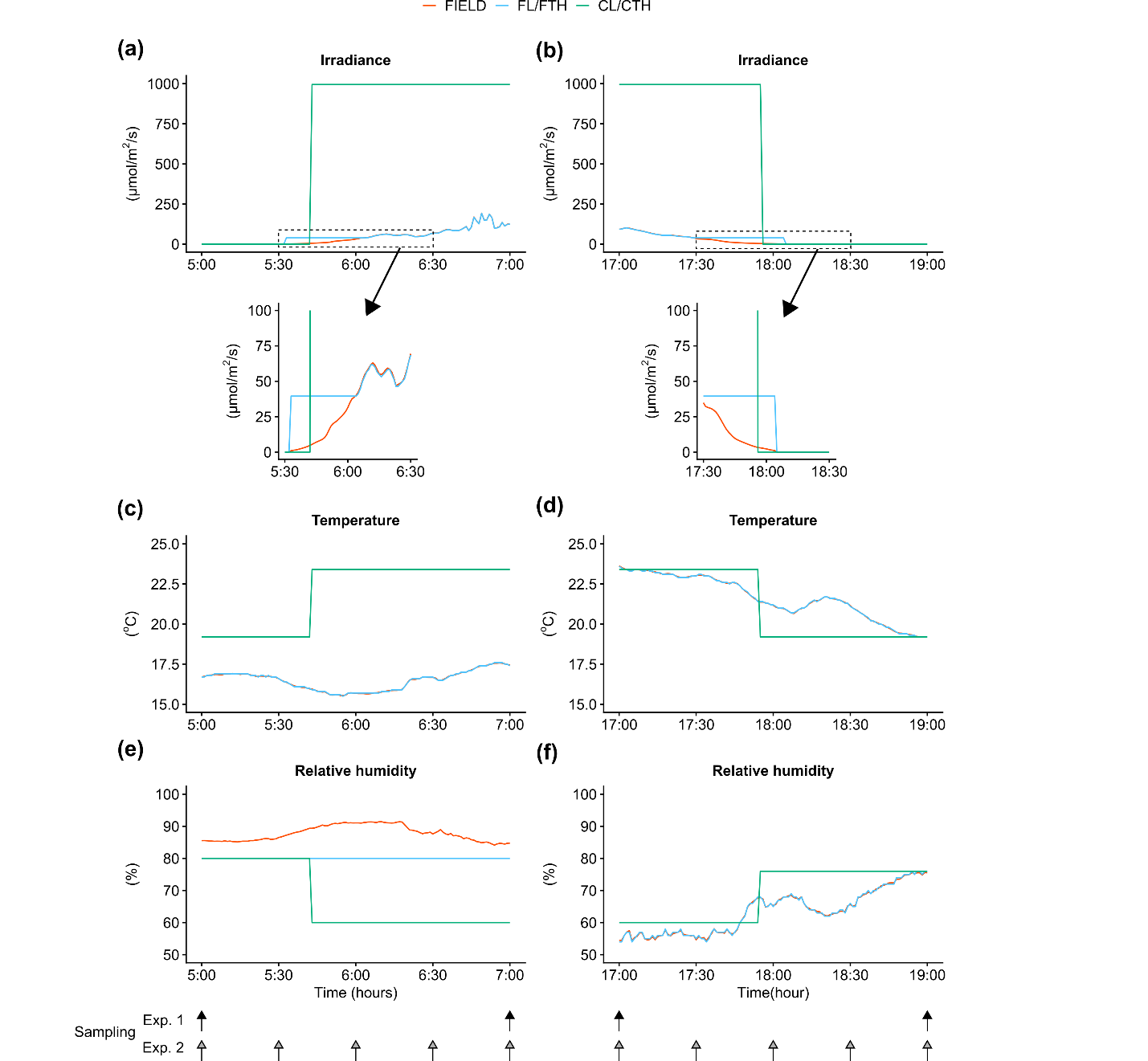


**Figure S4 Comparison of set and measured data of environmental conditions.** **(a, b)** Irradiance, **(c, d)** air temperature, and **(e, f)** relative humidity of **(a, c, e)** FL/FTH and **(b, d, f)** CL/CTH conditions. Sampling time-points are marked with arrows. The fluctuation of irradiance in FIELD was simulated in FL/FTH over three days, with the exception of the high irradiance in the sunny midday on the first and third days due to the upper limit of the light source output in SmartGC. Temperature was controlled by SmartGC in a range within 0.9 °C of the set value for FL/FTH and the values for the FIELD condition. Relative humidity was controlled in a range within 9% of the set value for FL/FTH and the values for the FIELD condition, except for high and low humidity (more than 80% and less than 50%) due to the upper and lower setting limit of SmartGC.


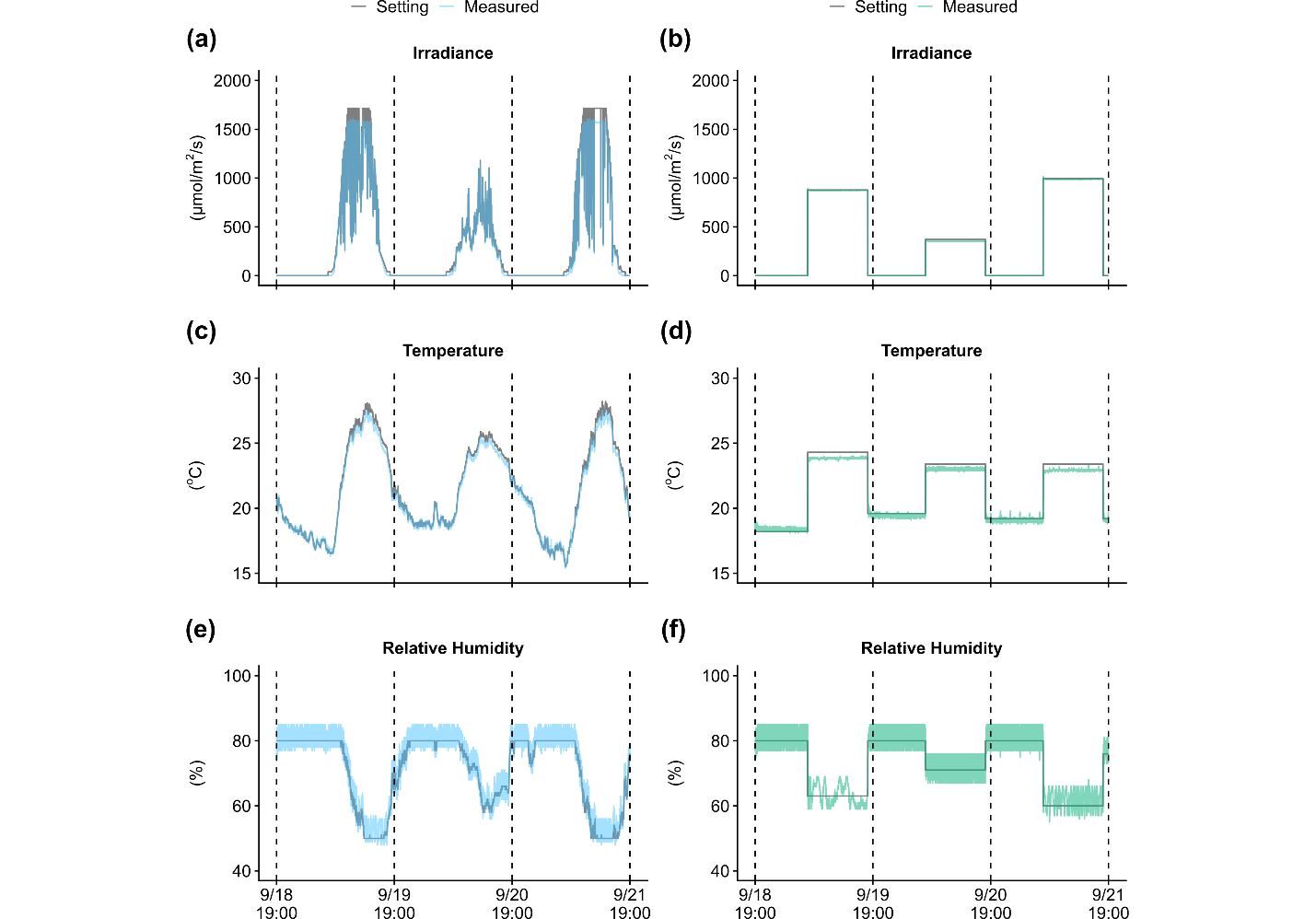
**Figure S5 Environmental conditions in the morning and evening on the sampling day.** **(a, b)** Irradiance, **(c, d)** air temperature, and **(e, f)** relative humidity from **(a, c, e)** 5:00–7:00 and **(b, d, f)** 17:00–19:00 on the sampling day measured in the FIELD condition and set for the FL/FTH and CL/CTH conditions. Sampling time-points are shown with arrows.


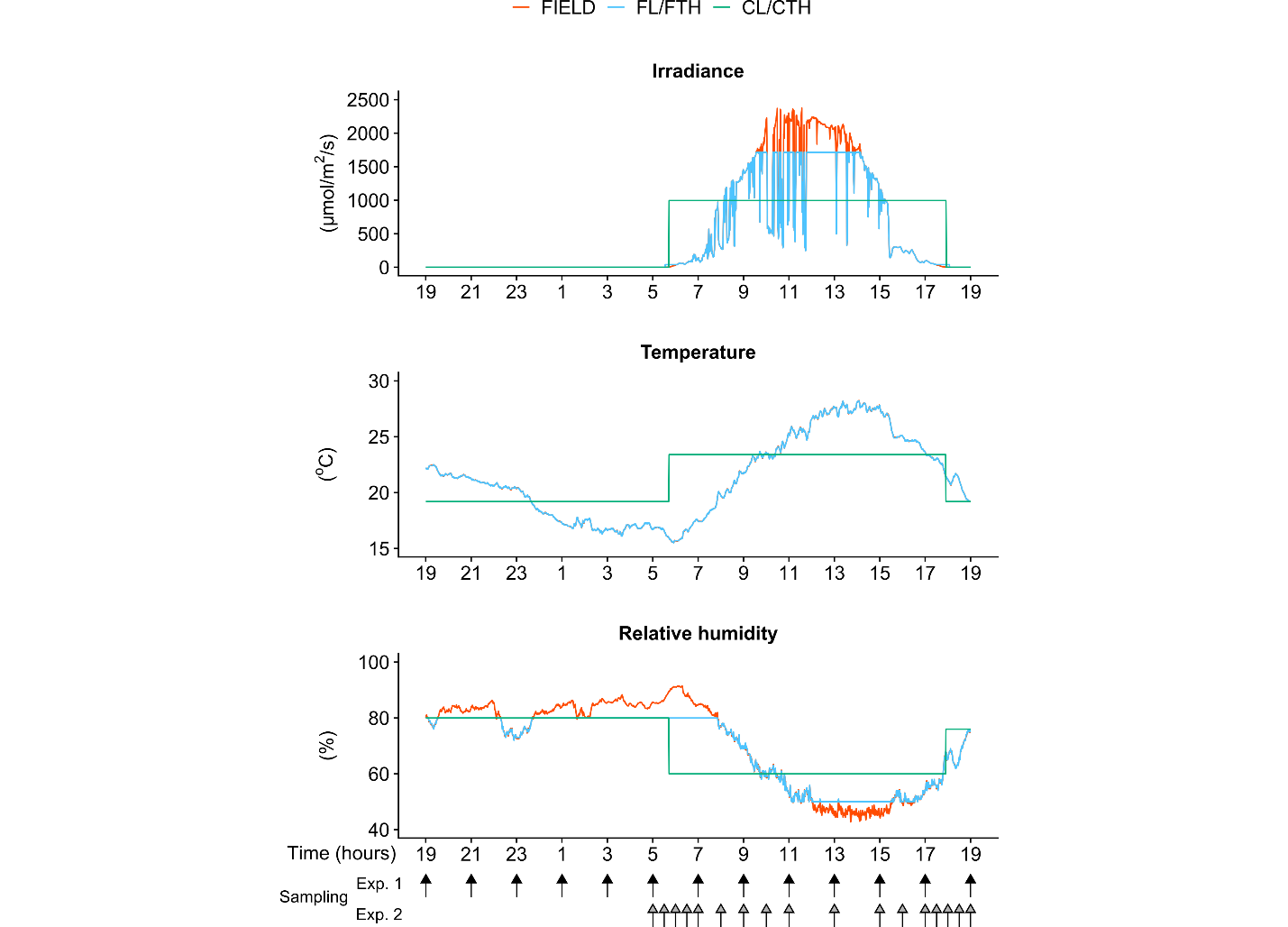
**Figure S6 Environmental conditions on the sampling day.** Sampling time-points are shown with arrows.


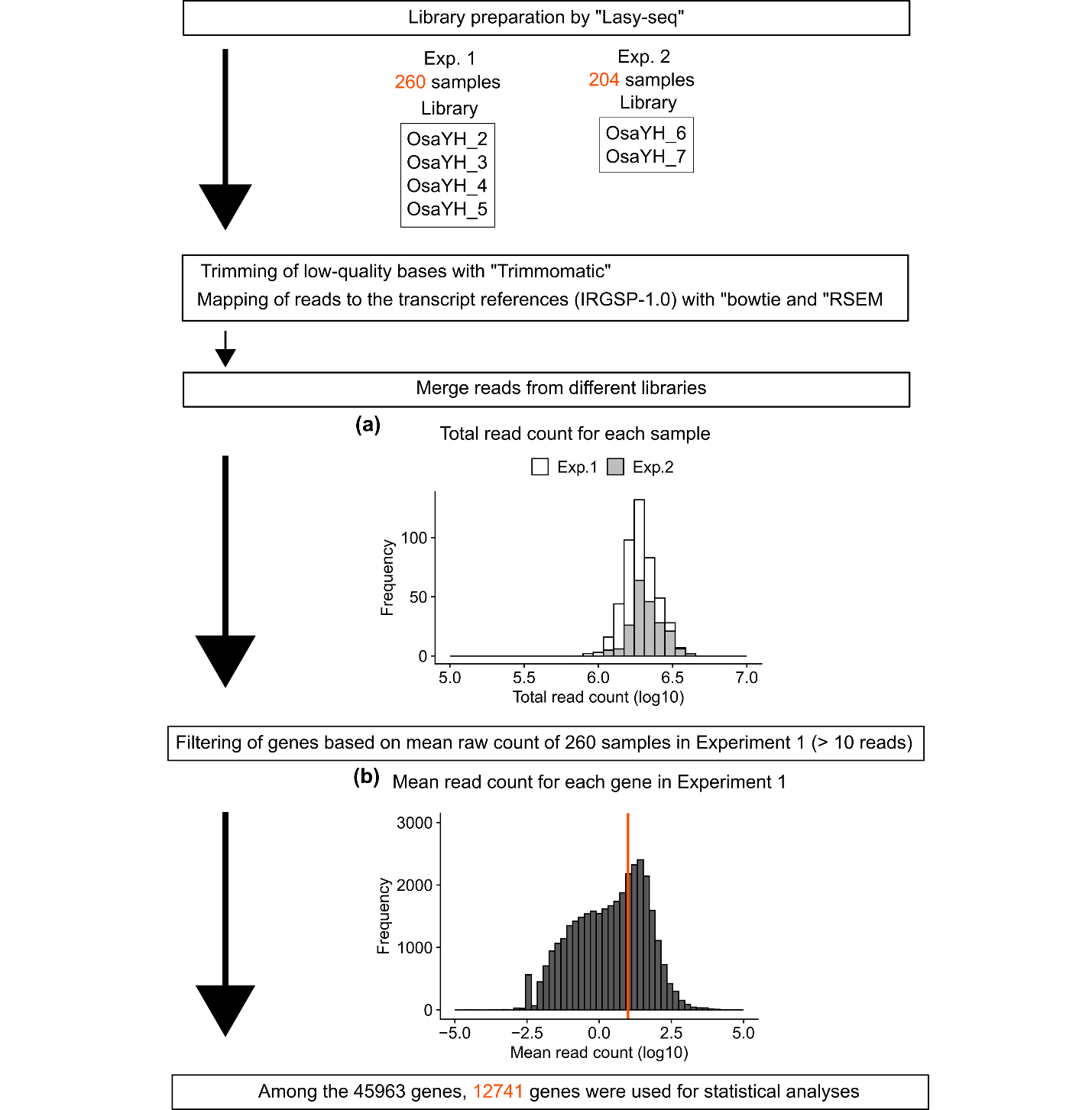
**Figure S7 Workflow of RNA-Seq data pre-processing. (a)** Histogram of the total read counts for each sample. Since some samples were sequenced using more than one library, reads for each sample were merged after mapping the reads to the transcript references. **(b)** Histogram of the mean read count for each gene in Experiment_1. After filtering, 12,741 genes were used for statistical analyses.


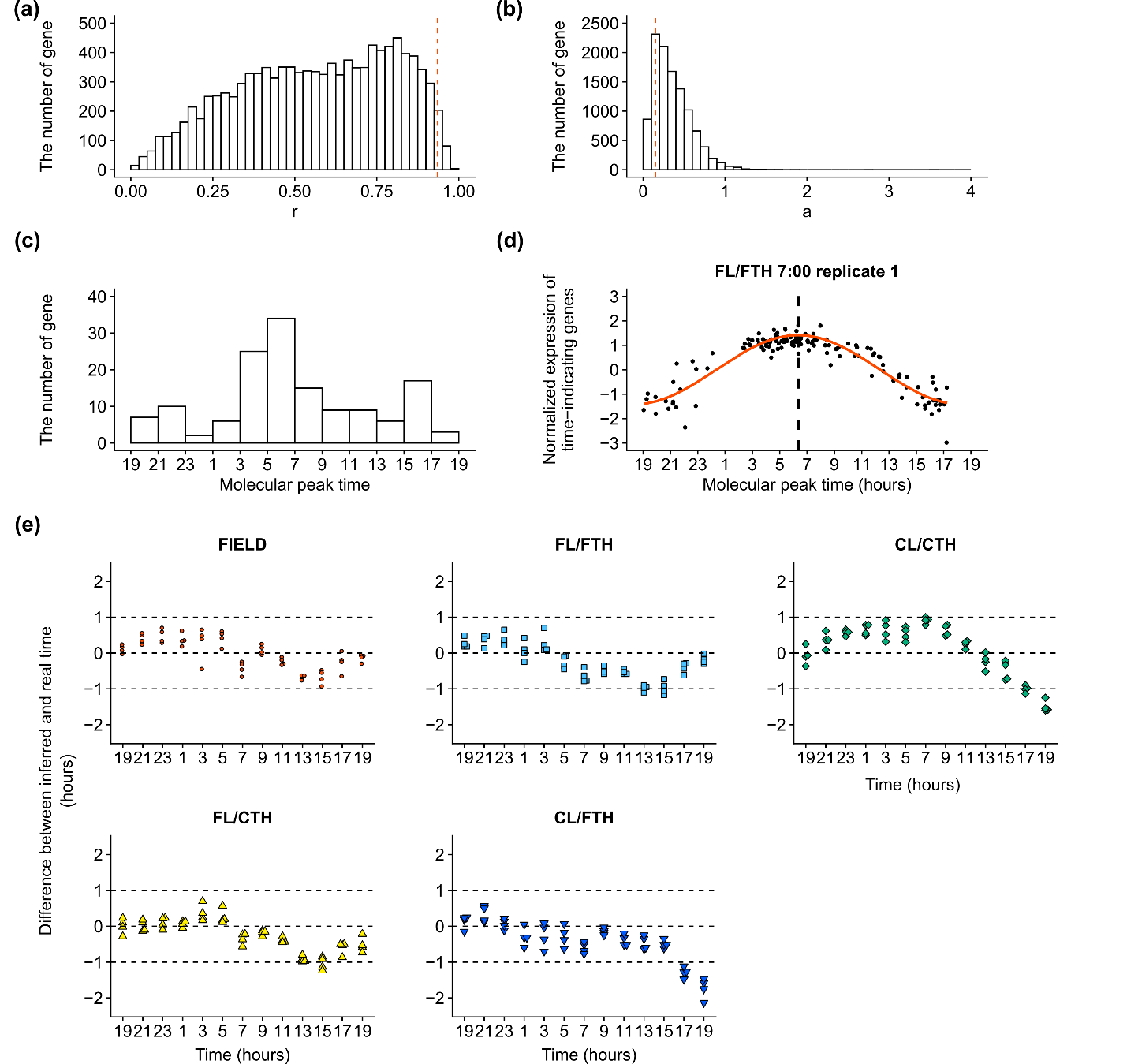
**Figure S8 Inference of internal time using the molecular timetable method.** **(a)** Histogram showing the Pearson correlation coefficient (r) between the expression of each gene and its best-fitting cosine curve. The red dashed line indicates the selection threshold (r = 0.935) for time-indicating genes. **(b)** Histogram showing the expression amplitude of each gene. The red dashed line indicates the selection threshold (a = 0.15) for time-indicating genes. **(c)** Histogram showing the molecular peak times of 143 time-indicating genes. **(d)** An example of internal time inference using the molecular timetable method. Normalized expression of time-indicating genes at the molecular peak time of each gene was fitted to the cosine curve, and the peak time indicates the internal time. **(e)** Inference of internal time at each condition in Experiment_1. Inferred time minus real time is shown for each sample (n = 4). Progression of internal time in the evening was slower in CL/CTH and CL/FTH than in FIELD.


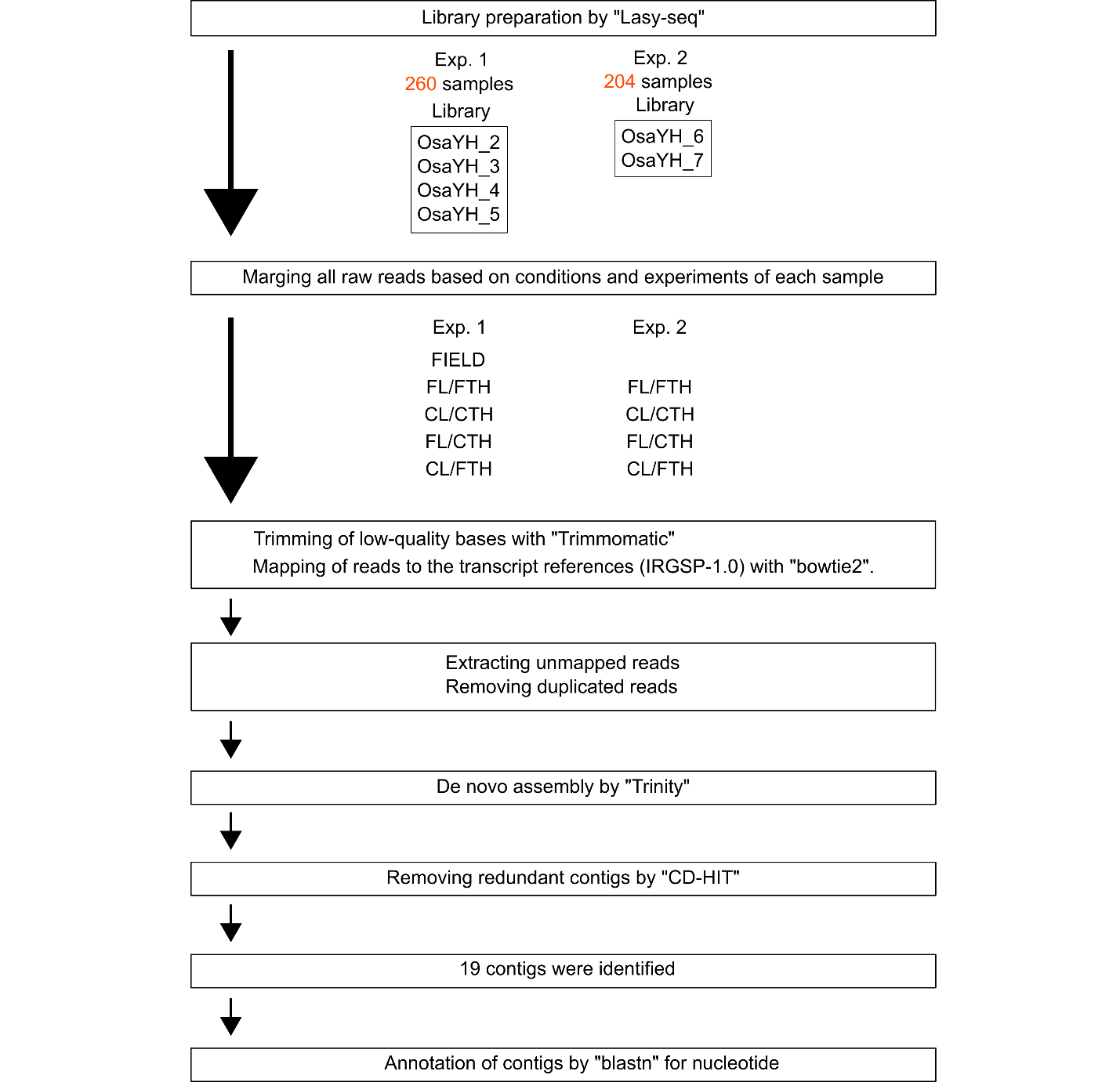
**Figure S9 Scheme for searching for genes that were found by de novo transcriptome assembly from unmapped reads to the rice reference transcriptomes.**


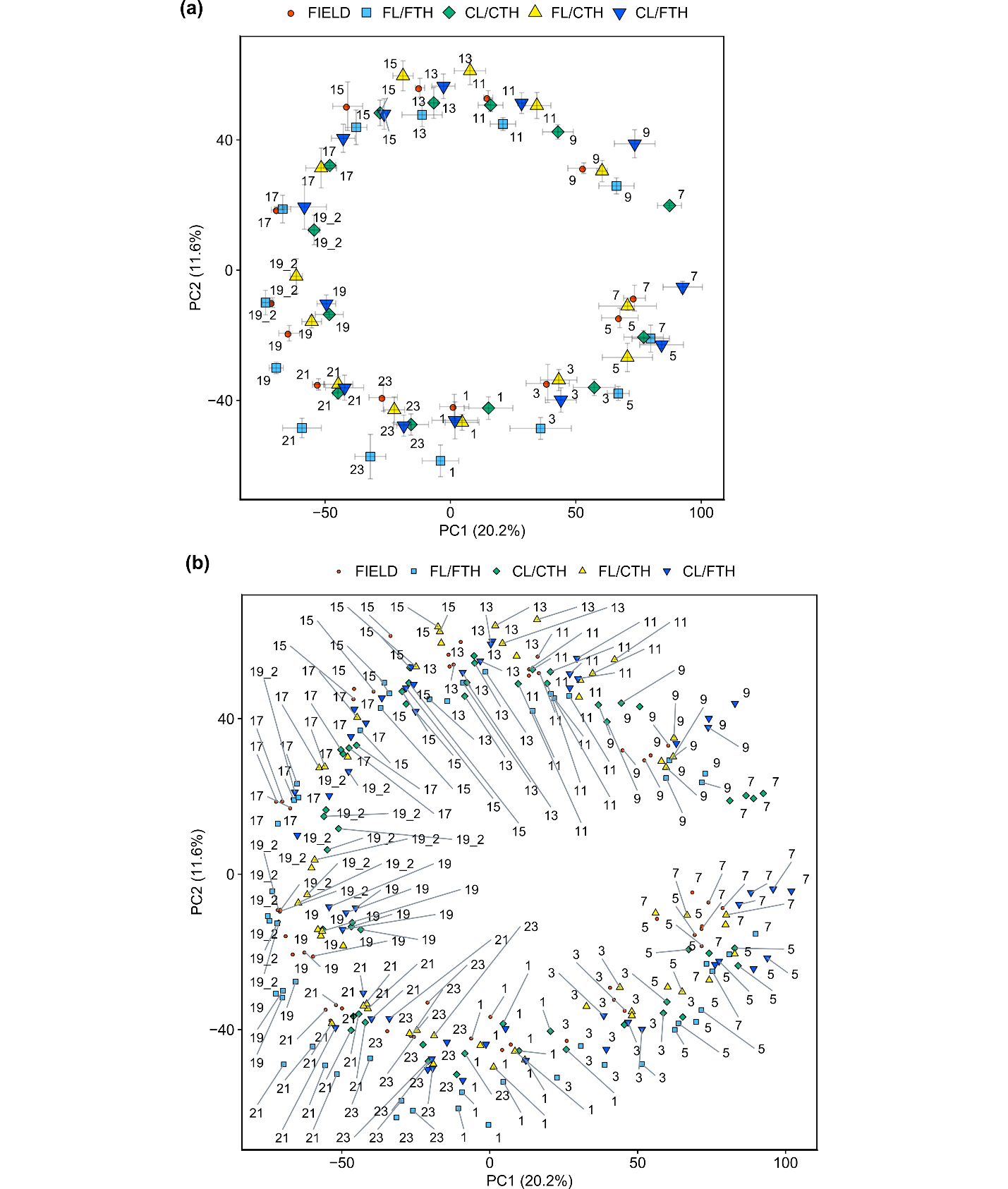
**Figure S10 Principal component analysis (PCA) of transcriptomes in Experiment_1.** **(a)** PCA of transcriptomes at each time-point and condition, which corresponds to Figure 1g. Each point represents the mean value of the four replicates, and error bars indicate the standard errors of PC1 and PC2. **(b)** PCA of transcriptomes of each sample at each time-point and condition. Four replicates at each time-point and condition are shown independently. Numbers indicate sampling times. The percentages of the total variance represented by PC1 and PC2 are shown in parentheses. 19_2 indicates the time-point 24 h after the start of sampling day at 19:00.

**
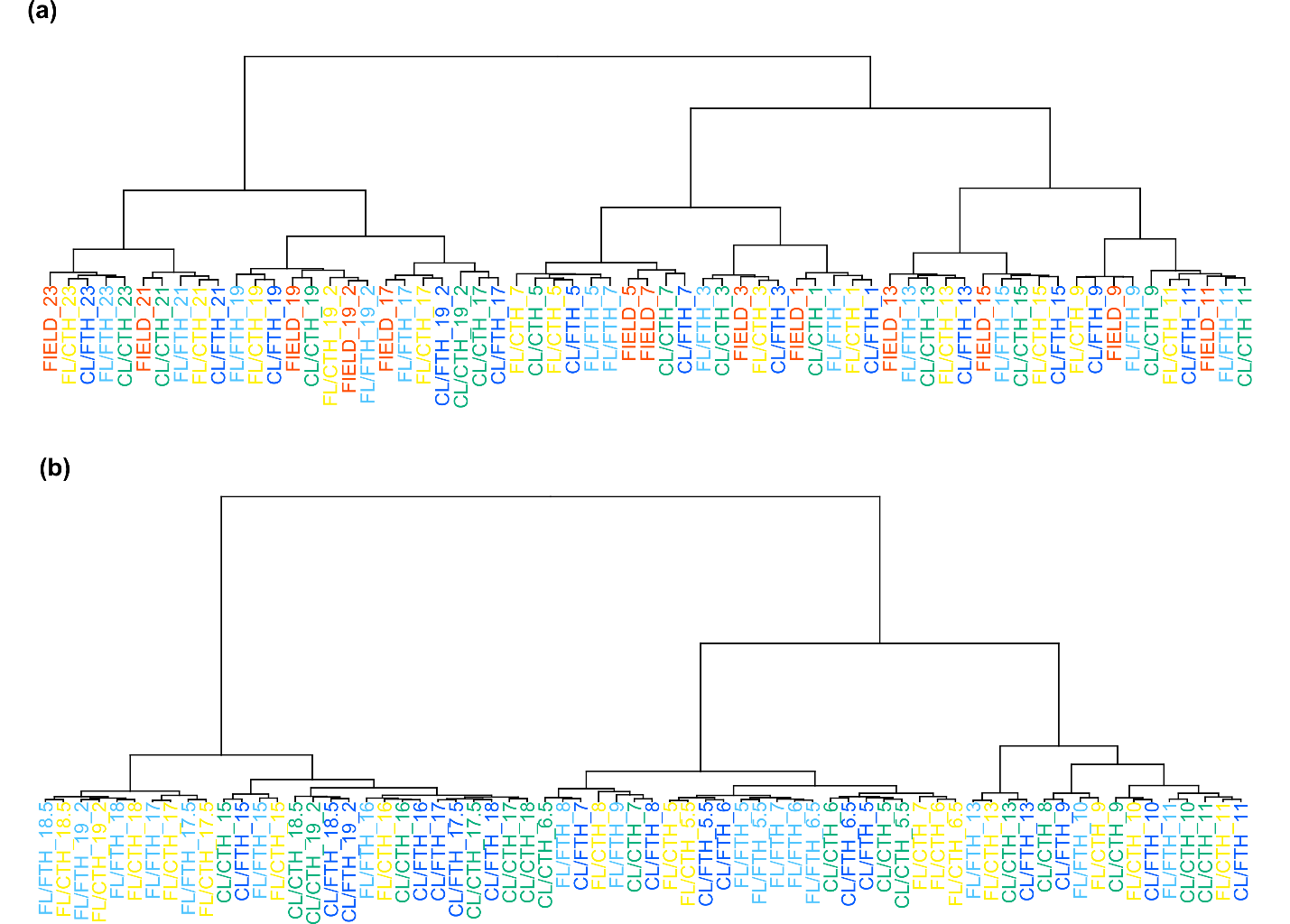
Figure S11 Hierarchical cluster dendrogram of transcriptomes of each condition and time-point.** The dendrograms in Figure 1i and Figure 1j correspond to **(a)** and **(b)**, respectively. Mean values of **(a)** 4 and **(b)** 3 replicates at each time-point and condition were used for the analyses. 19_2 indicates the time-point 24 h after the start of sampling day at 19:00.


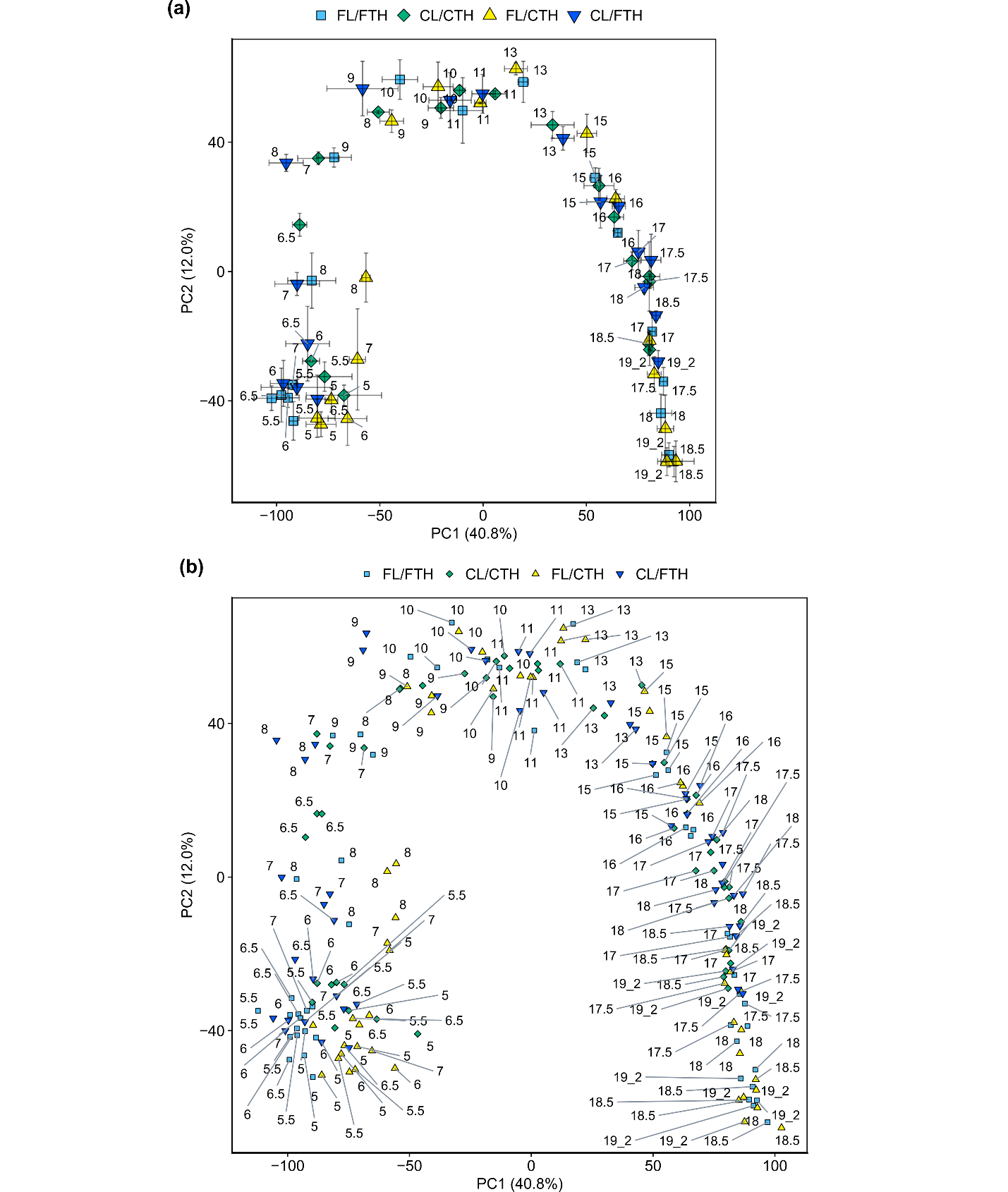
**Figure S12 Principal component analysis (PCA) of transcriptomes in Experiment_2.** **(a)** PCA of transcriptomes at each time-point and condition, which correspond to Figure 1h. Each point represents the mean value of three replicates, and error bars indicate standard errors of PC1 and PC2. **(b)** PCA of transcriptomes of each sample at each time-point and condition. Four replicates at each time-point and condition are shown independently. Numbers indicate sampling times. The percentages of total variance represented by PC1 and PC2 are shown in parentheses. 19_2 indicates the time-point 24 h after the start of sampling day at 19:00.


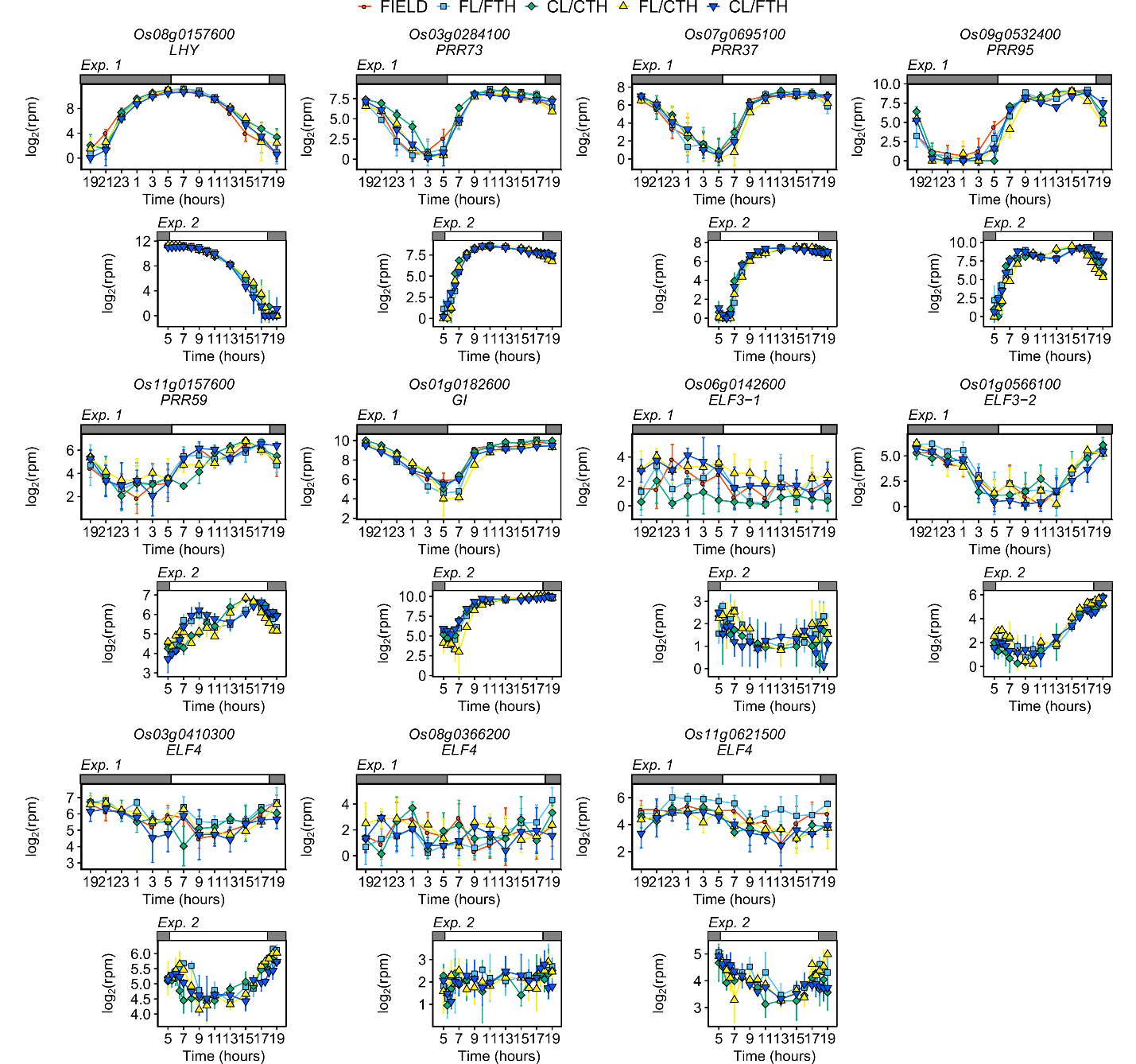
**Figure S13 Expression of genes encoding core circadian clock genes.** Points indicate means, and error bars indicate standard deviations (n = 4 and n = 3 in Experiment_1 and Experiment_2, respectively). *LHY*, *LATE ELONGATED HYPOCOTYL*; *PRR73*, *PSEUDO-RESPONSE REGULATOR 73*; *PRR37*, *PSEUDO-RESPONSE REGULATOR 37*; *PRR95*, *PSEUDO-RESPONSE REGULATOR 95*; *PRR59*, *PSEUDO-RESPONSE REGULATOR 59*; *GI*, *GIGANTIA*; *ELF3, EARLY-FLOWERING 3*; *ELF4*, *EARLY-FLOWERING 4*.


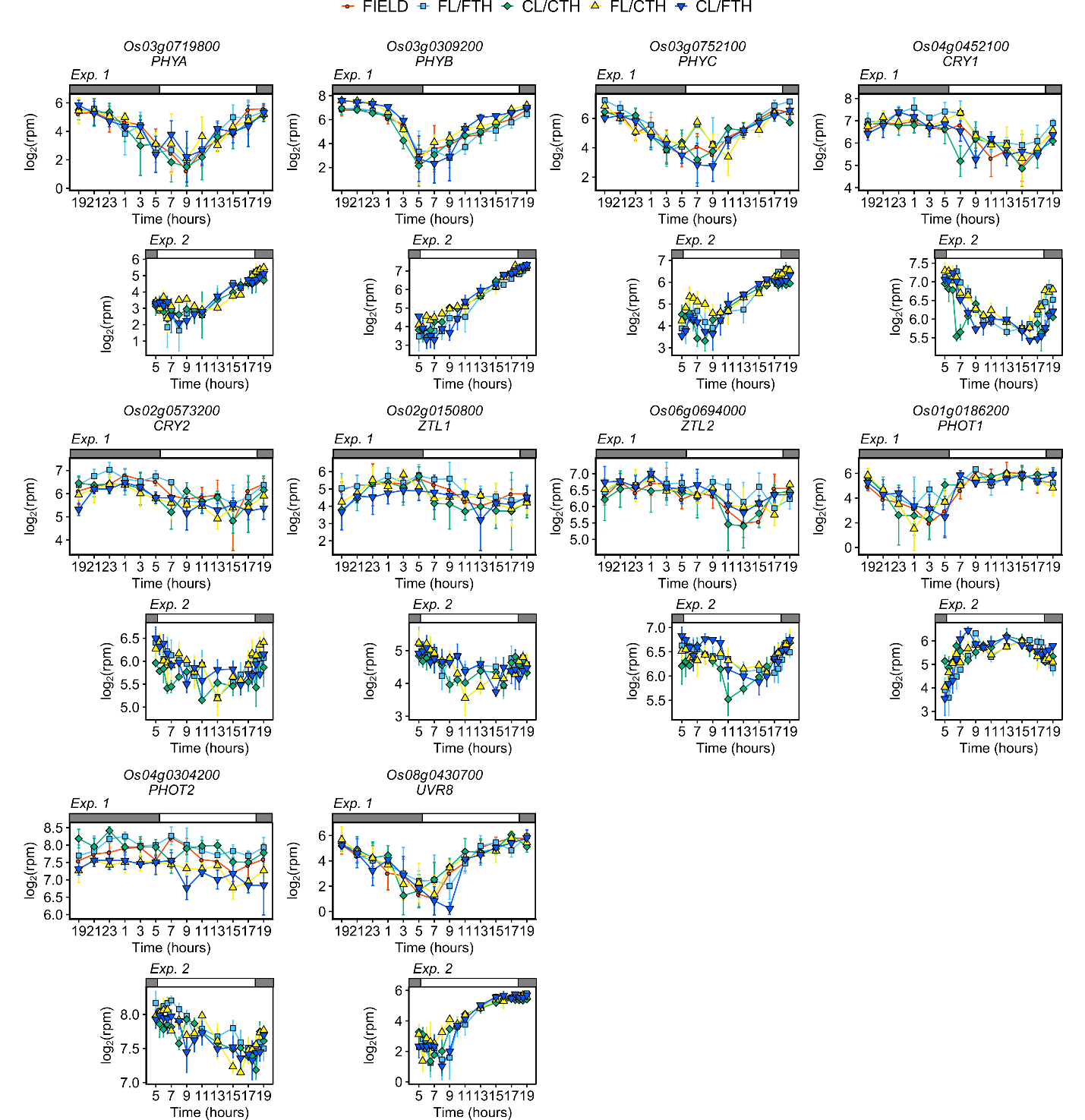
**Figure S14 Expression of genes encoding photoreceptors.** Points indicate means, and error bars indicate standard deviations (n = 4 and n = 3 in Experiment_1 and Experiment_2, respectively). *CRY*, cryptochrome; *PHOT*, phototropin; *PHY*, phytochrome; *UVR8*, UV-B resistance 8; *ZTL*, *ZEITLUPE*.


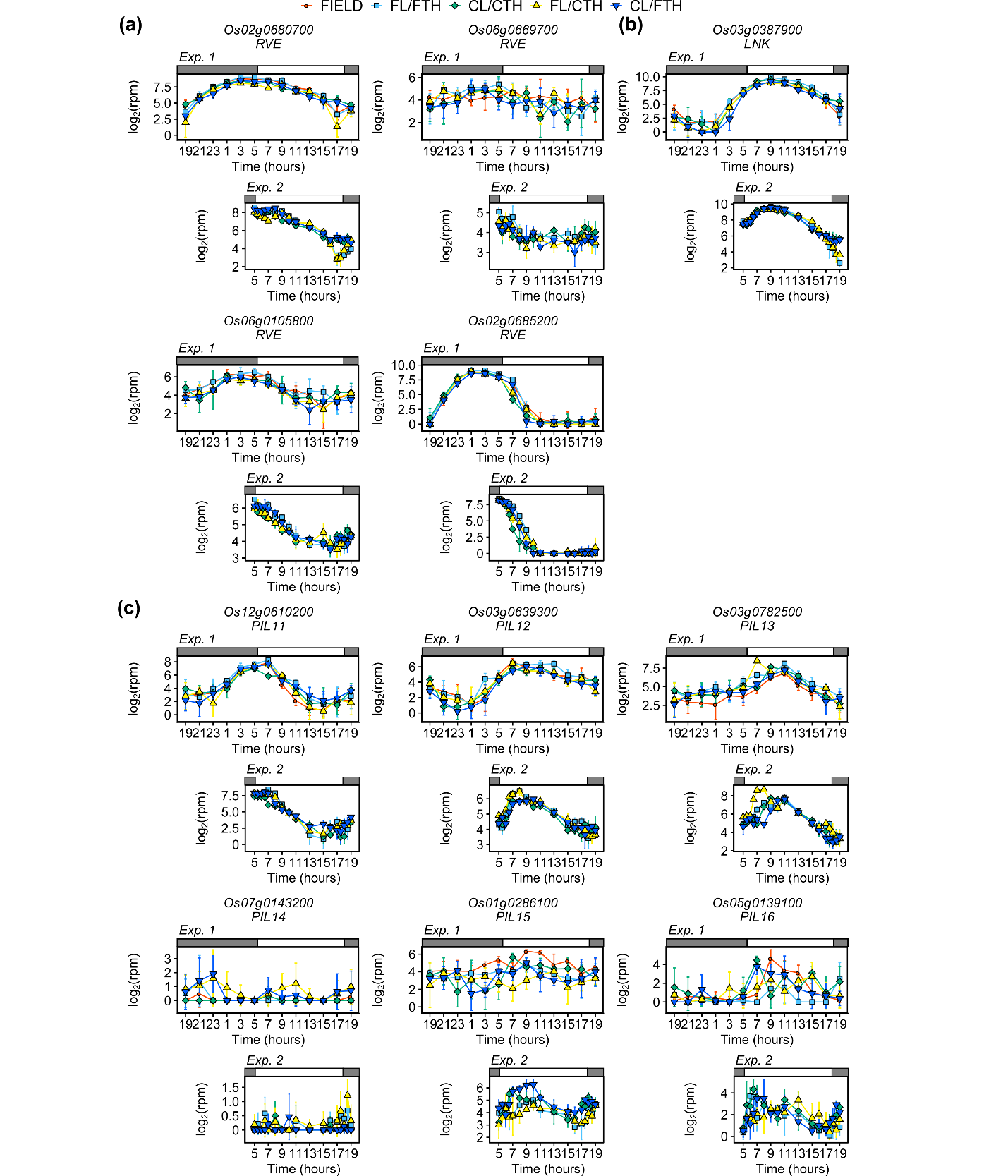
**Figure S15 Expression of genes encoding (a) REVEILLE (RVE), (b) NIGHT LIGHT-INDUCIBLE AND CLOCK-REGULATED protein (LNK), and (c) PHYTOCHROME INTERACTING FACTOR-LIKE protein (PIL).** Points indicate means, and error bars indicate standard deviations (n = 4 and n = 3 in Experiment_1 and Experiment_2, respectively).


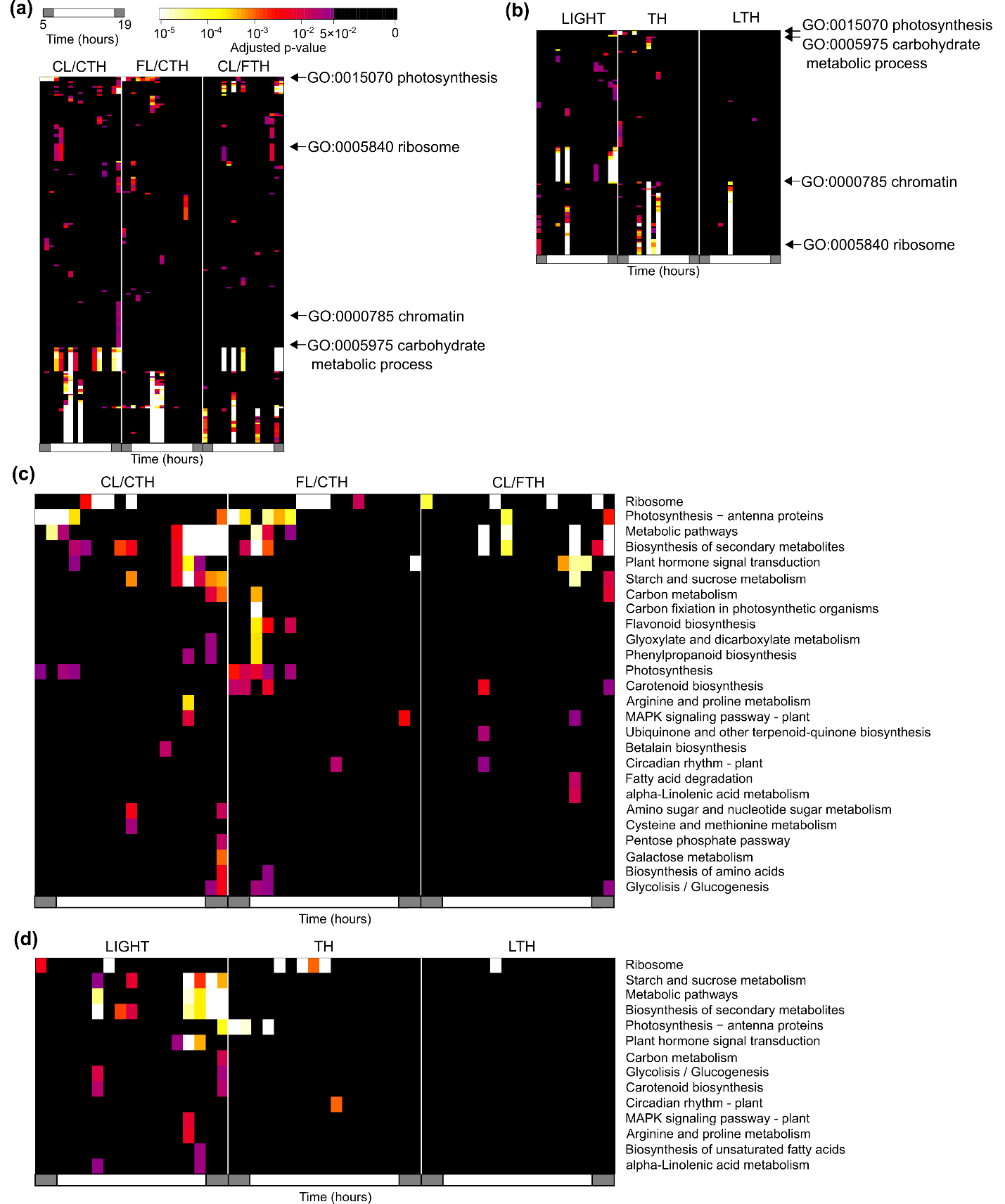
**Figure S16 Significant enrichment of genes with specific annotations in DEGs between FIELD and the other conditions in Experiment_2.** Heatmaps of P-values (Fisher’s exact test, two-sided) for significant genes with **(a, b)** a particular gene ontology (GO) and **(c, d)** a particular KEGG pathway (row) in each time and condition (column) in **(a, c)** DEGs between FIELD and the other conditions and **(b, d)** LIGHT, TH, LTH, and UNREP genes. GO and KEGG pathways that have at least one significant (adjusted p-value < 0.05) time-point and condition are shown.


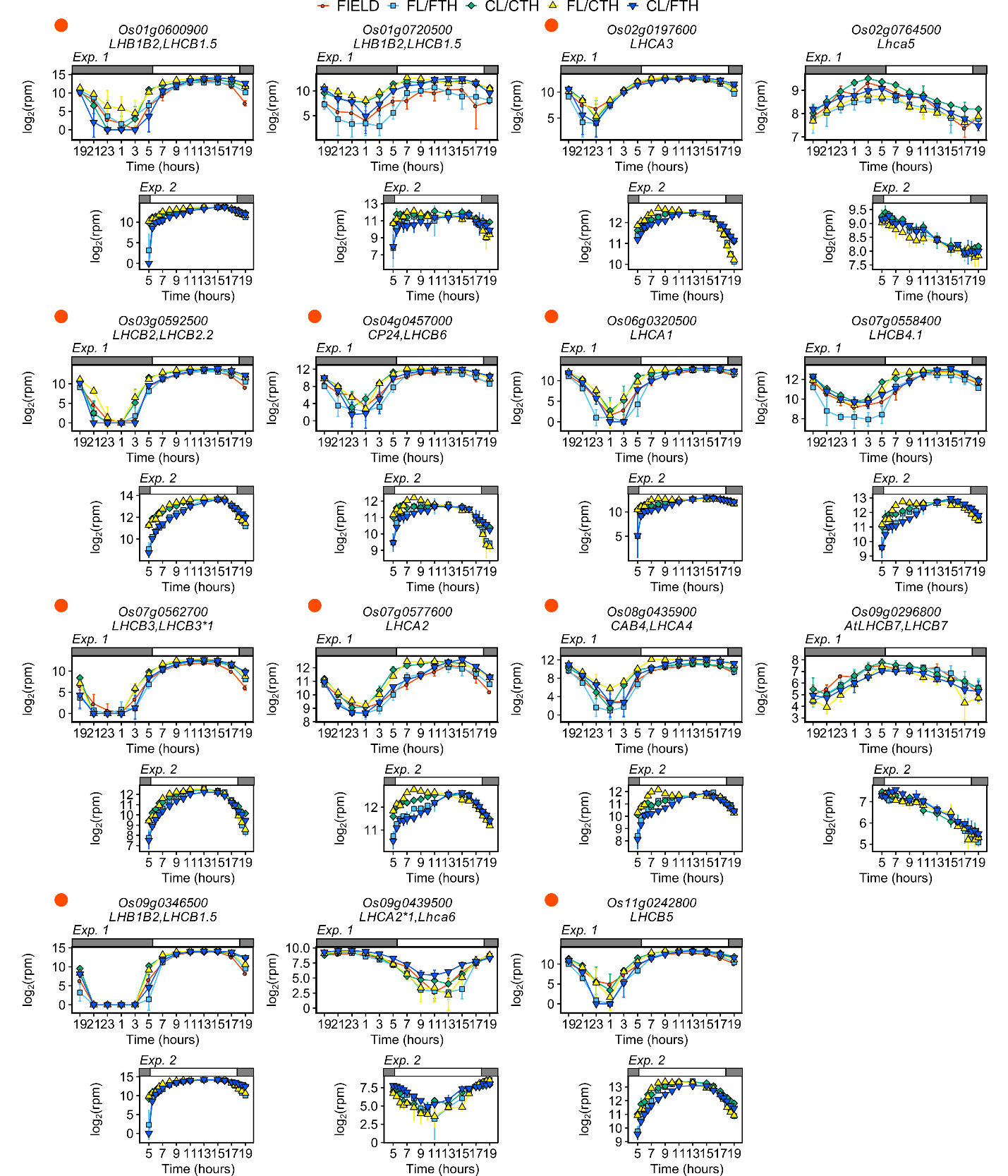
**Figure S17 Expression of genes related to light harvesting.** Expression of genes with annotation for photosynthesis-antenna protein (KEGG pathway: dosa00196) or photosynthetic light harvesting (GO:0009765) in Experiment_1 and Experiment_2 is shown. Red points on the left side of the gene name indicate that the gene was affected by night temperature in Nagano et al. (2012) (See Manuscript and Table S14). Points indicate means, and error bars indicate standard deviations (n = 4 and n =3 in Experiment_1 and Experiment_2, respectively).


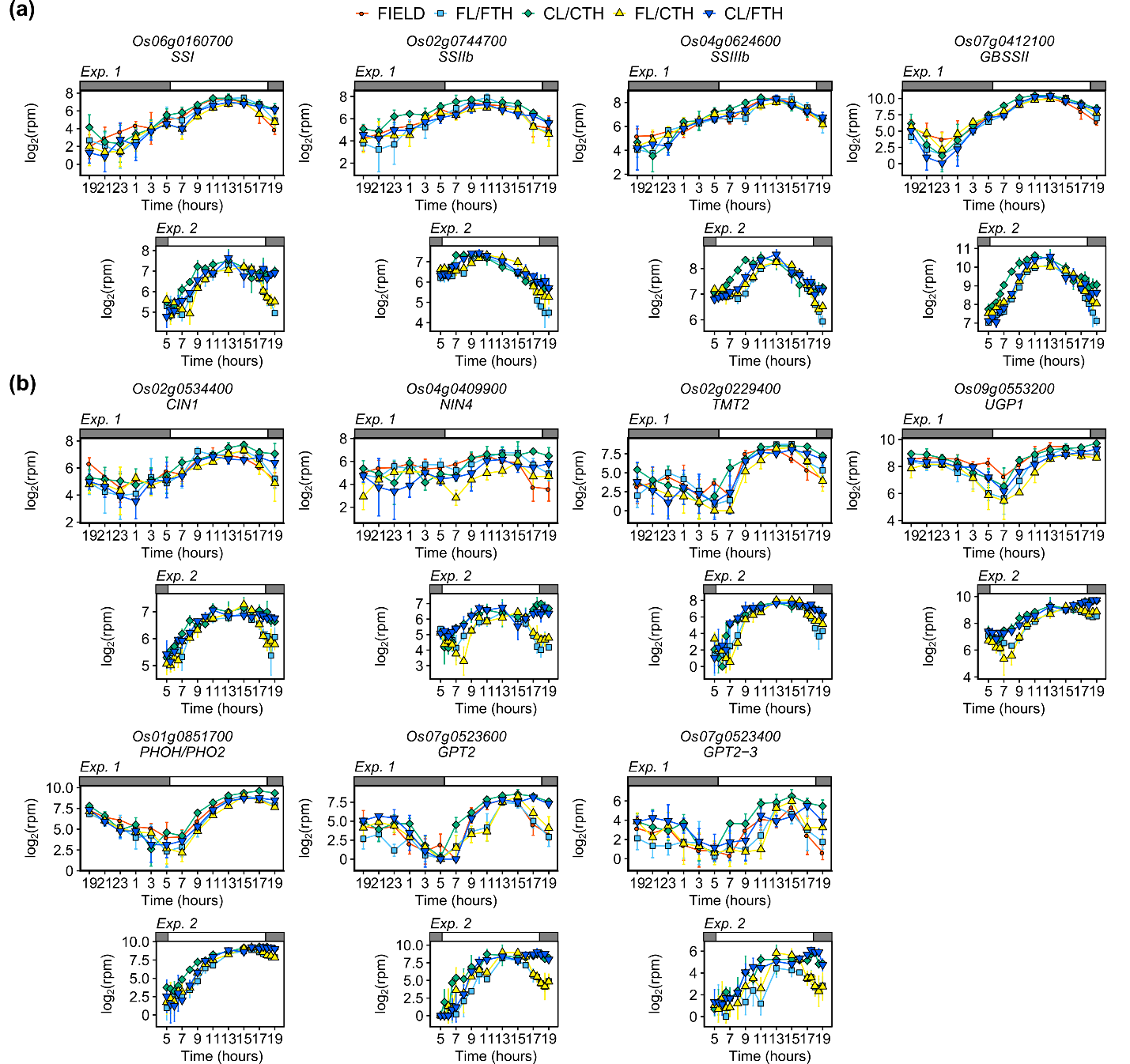
**Figure S18 Expression of genes related to (a) starch and (b) sucrose metabolism.** Points indicate means, and error bars indicate standard deviations (n = 4 and n = 3 in Experiment_1 and Experiment_2, respectively). *CIN1*, cytosolic invertase 1; *GPT2*, glucose-6-phosphate/phosphate translocator 2; *GPT2-3*, glucose-6-phosphate/phosphate translocator 2-3; *GBSSII*, Granule-bound starch synthase II; *NIN4*, neutral invertase 4; *PHOH/PHO2*, starch phosphorylase 2; *SSI*, starch synthase I; *SSIIb*, starch synthase IIb; *SSIIIb*, starch synthase IIIb; *TMT2*, tonoplast monosaccharide transporter 2; *UGP1*, UDP-glucose pyrophosphorylase 1.


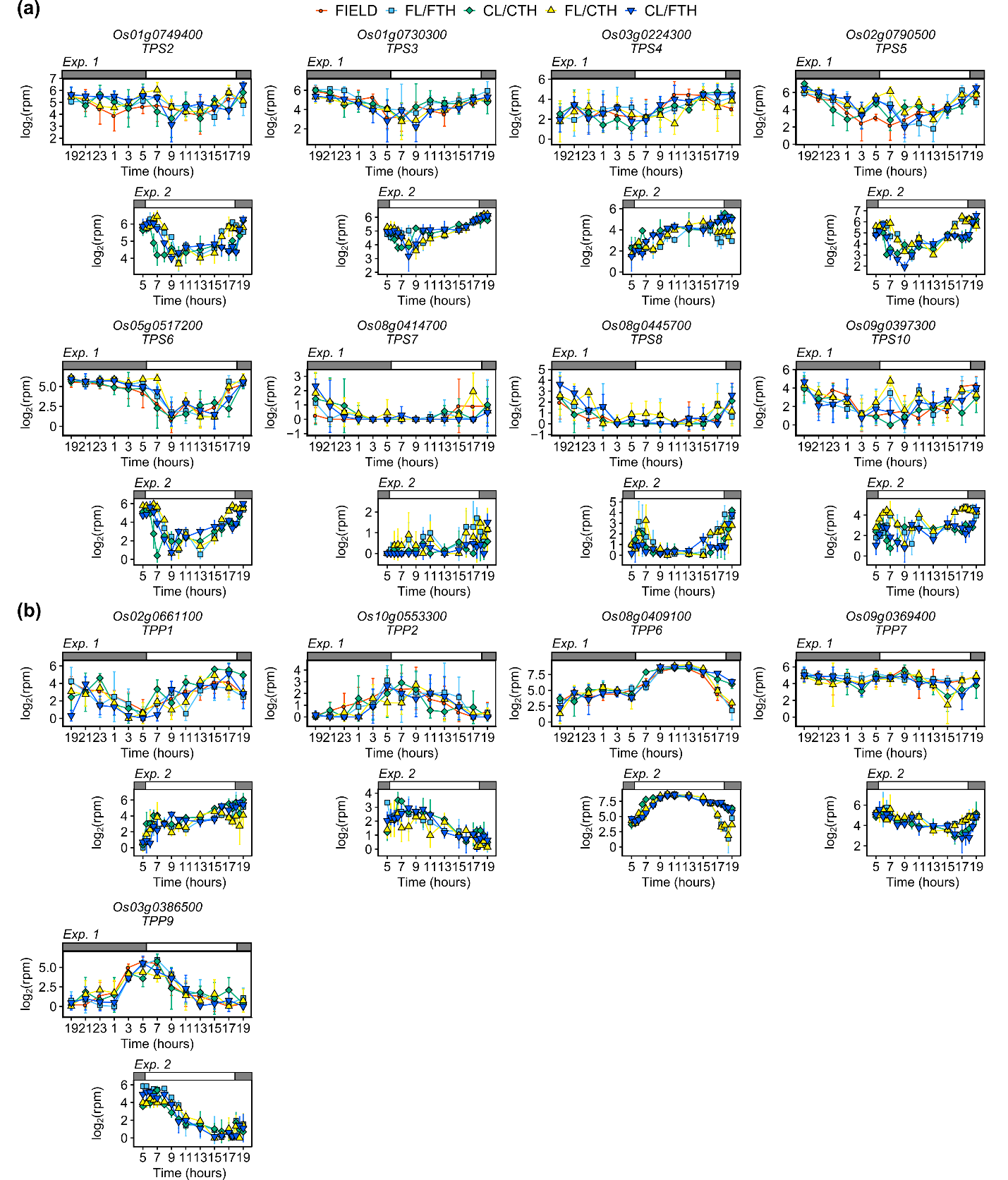
**Figure S19 Expression of genes encoding (a) trehalose phosphate synthase (TPS) and (b) trehalose phosphate phosphatase (TPP).** Expression of *TPS1* is shown in Figure 5e. *TPS9*, *TPS11*, *TPP3*, *TPP4*, *TPP5*, and *TPP8* are not shown because of their low expression. Points indicate means, and error bars indicate standard deviations (n = 4 and n = 3 in Experiment_1 and Experiment_2, respectively).


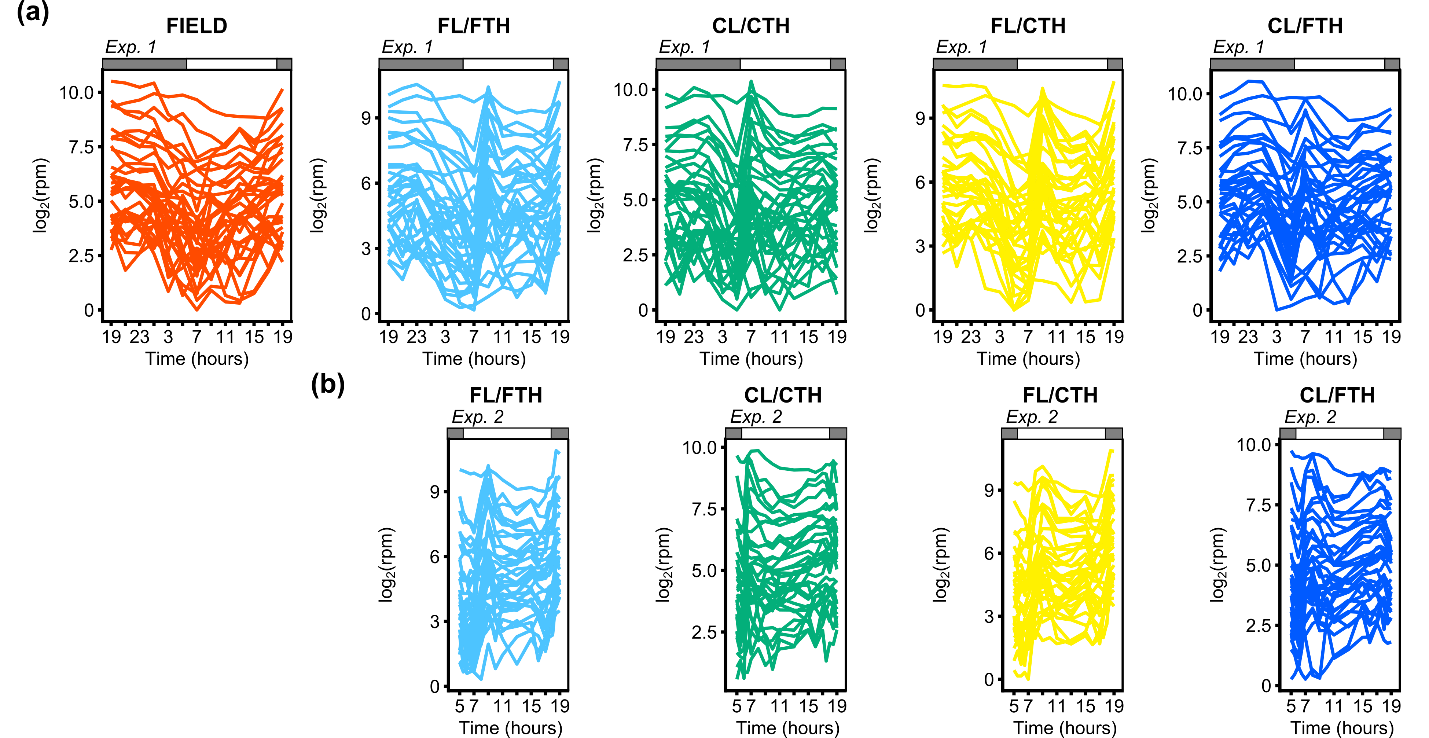
**Figure S20 Different timing of upregulation of chromatin-related genes between conditions.** Expression of genes with annotations for chromatin (GO:0000785) in **(a)** Experiment_1 and **(b)** Experiment_2. Each line indicates the mean value of the expression of each gene (n = 4 and n = 3 in Experiment_1 and Experiment_2, respectively). Expression of genes with annotation for chromatin was induced at 5:00–7:00 in CL/CTH and CL/FTH, and at 7:00–9:00 in FL/FTH and FL/CTH, but the induction in the morning was not obvious in FIELD. The timing of induction was reproduced in Experiment_2. These results suggest that the expression of genes with annotation for chromatin was induced by LED light, but not by sunlight, in the morning, and the timing of induction was affected by diurnal changes in irradiance.


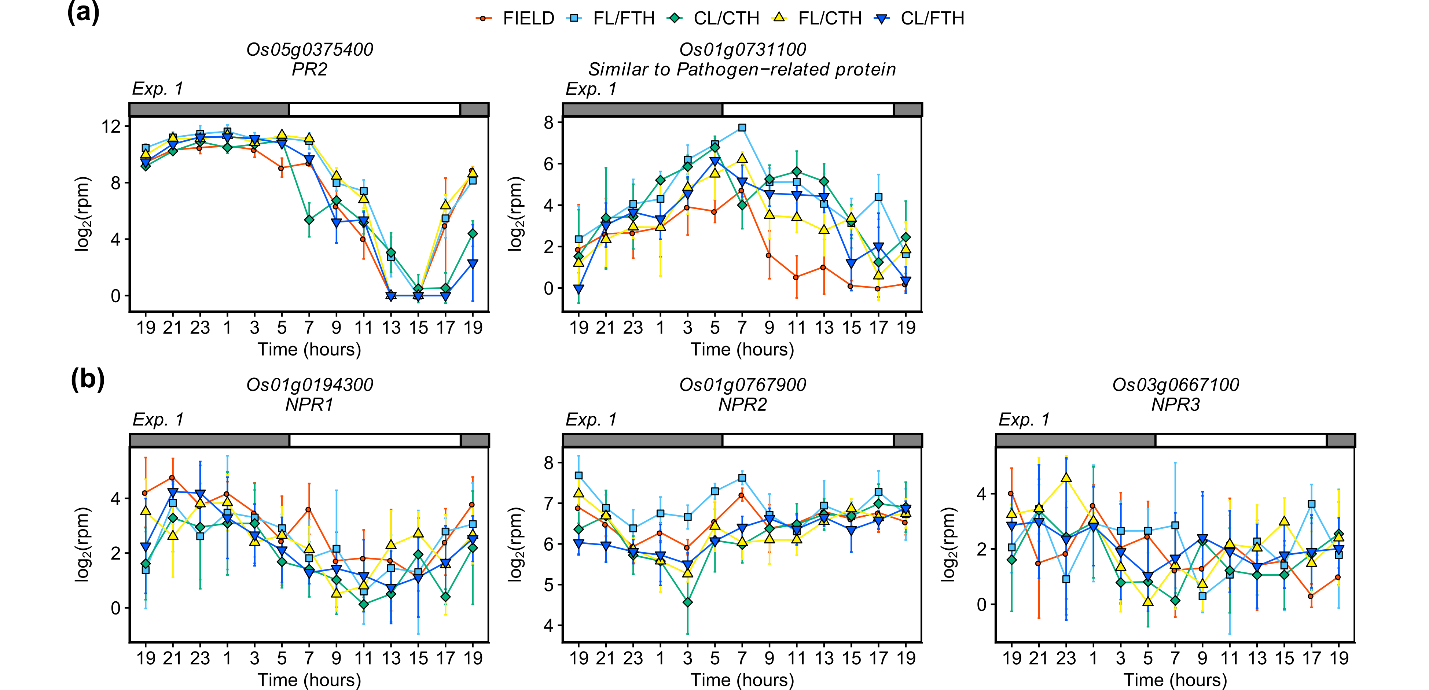
**Figure S21 Expression of (a) pathogenesis-related (PR) genes and (b) NONEXPRESSOR OF PATHOGENESIS-RELATED (NPR) GENES in Experiment_1.** Points indicate means, and error bars indicate standard deviations (n = 4).


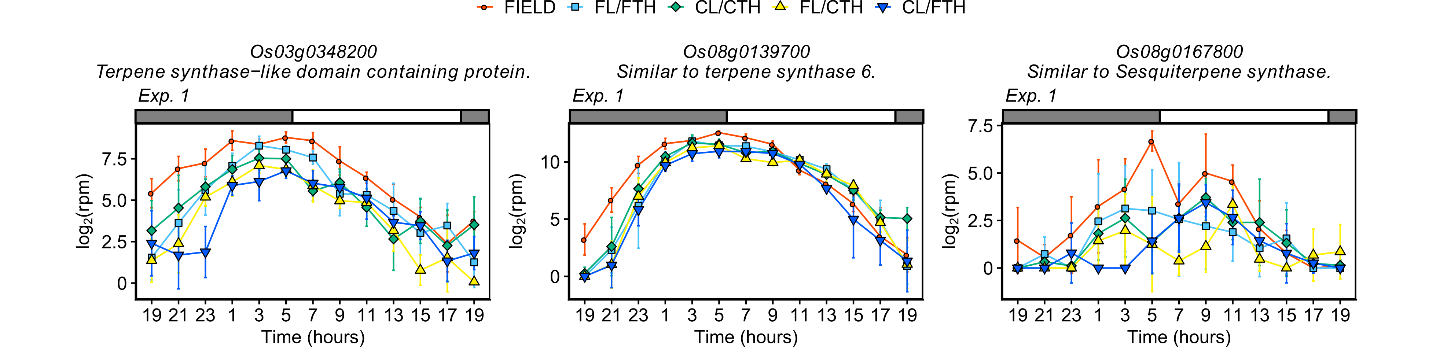
**Figure S22 Expression of genes related to terpene synthase in Experiment_1.** Points indicate means, and error bars indicate standard deviations (n = 4).


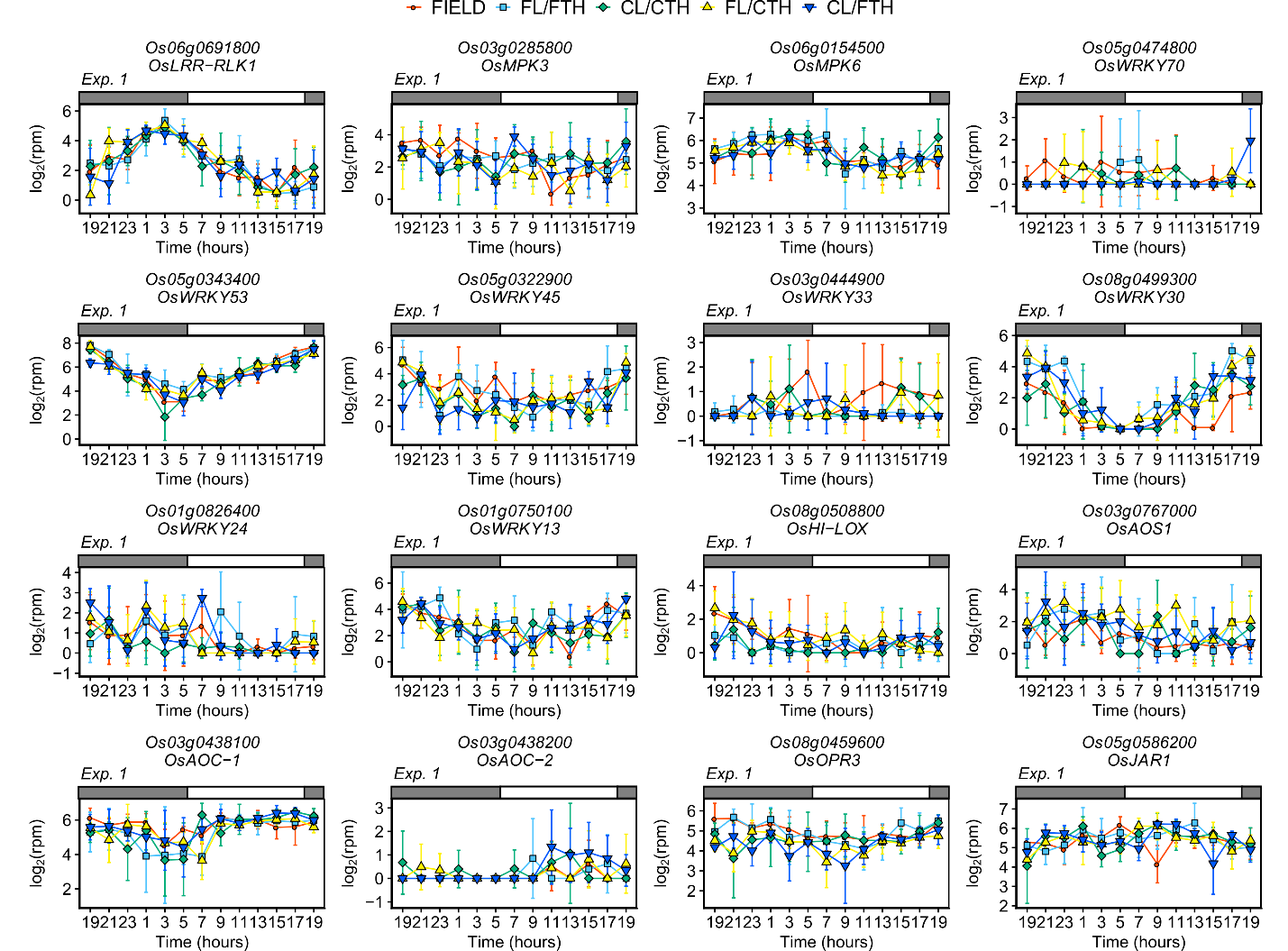
**Figure S23 Expression of genes involved in early defense signaling to herbivore attack listed in Ye et al. (2019) in Experiment_1.** Points indicate means, and error bars indicate standard deviations (n = 4). Early defense signaling genes include *Oryza sativa* leucine-rich repeat receptor-like kinase 1 (*OsLRR-RLK1*), mitogen-activated protein kinase (*OsMPK3* and *OsMPK6*), WRKY transcription factors (*OsWRKY13*, *OsWRKY24*, *OsWRKY30*, *OsWRKY33*, *OsWRKY45*, *OsWRKY53*, and *OsWRKY70*), and jasmonate synthesis genes (*OsHI-LOX*, *OsAOS1*, *OsAOC*, *OsOPR3*, and *OsJAR1*).


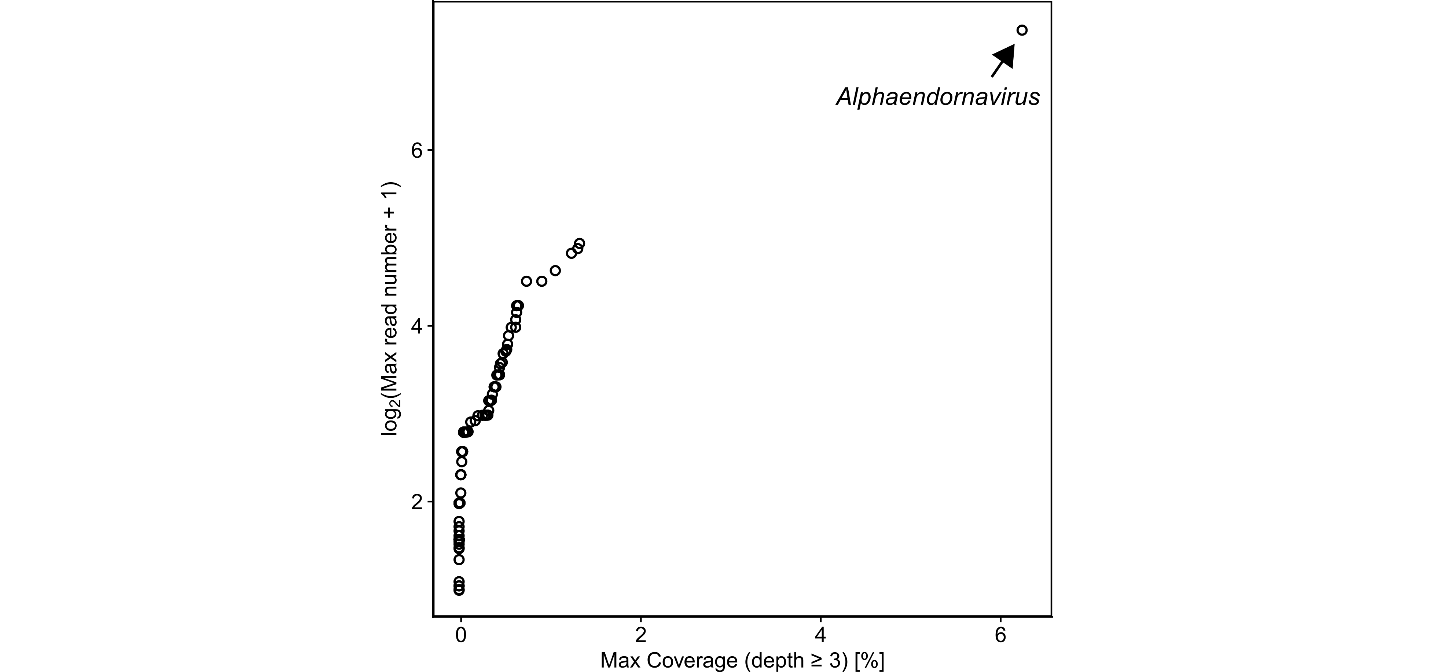
**Figure S24 Detection of virus infection from RNA-Seq data.** Scatter plot showing the max read number and max coverage of 115 viruses with maximum reads per sample >1. *Alphaendornavirus*, which shows no clear symptoms in rice plants (Moriyama et al., 1995), had the highest reads (maximum 166 reads per sample) and coverage of the reads to the reference (6.3% with depth ≥ 3). Correlations between the read number of the *Alphaendornavirus* and the expression of PR genes are shown in Figure 6f. The number of reads and the coverage of the *Alphaendornavirus* were low in comparison with infected samples discussed in Kamitani et al. (2016) (more than 10% in a sample with the lowest coverage). Therefore, this result suggests that even if *Alphaendornavirus* existed in the rice leaves, the copy number of the virus was limited.


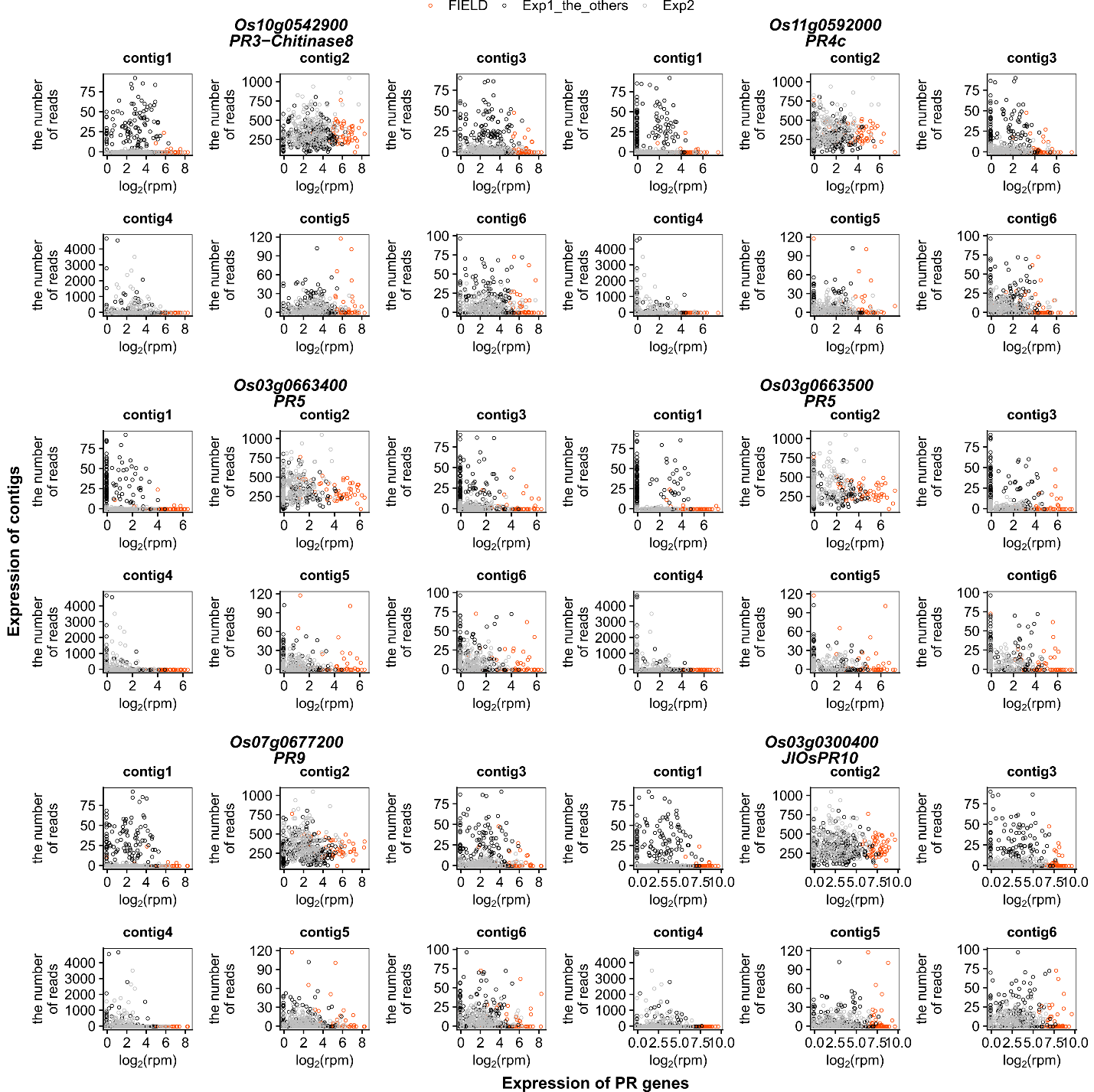
**Figure S25 Scatter plot showing the relationship between PR genes and contigs found by de novo transcriptome assembly from unmapped reads to the rice reference transcriptomes.** We selected 6 contigs from 19 contigs after excluding those with annotations for *Alphaendornavirus*, synthetic constructs, and expression vectors. contig1, exp1_CL/FTH.TRINITY_DN138_c0_g1_i1; contig2, exp1_FL/CTH.TRINITY_DN11_c0_g1_i1; contig3, exp1_FL/CTH.TRINITY_DN249_c0_g1_i2; contig4, exp2_CL/CTH.TRINITY_DN586_c0_g1_i1; contig5, exp2_CL/FTH.TRINITY_DN544_c0_g3_i1; contig6, exp2_FL/CTH.TRINITY_DN119_c0_g1_i1. Details of each gene are shown in Table S3.

**Supporting Information Tables**

**Table S1 Sample attributes used in this study.**

**Table S2 Time-indicating genes used in this study.**

**Table S3 List of contigs identified by de novo transcriptome assembly from unmapped reads to the rice reference transcriptome.**

**Table S4 The q-value of DEG analysis between FIELD and other conditions at each time -point in Experiment_1.**

**Table S5 The q-value of DEG analysis between FL/FTH and other conditions at each time-point in Experiment_2.**

**Table S6 Enriched gene ontology in DEGs between FIELD and other conditions in Experiment_1.**

**Table S7 Enriched gene ontology in LIGHT, TH, LTH, and UNREP genes in Experiment_1.**

**Table S8 Enriched KEGG pathway in DEGs between FIELD and other conditions in Experiment_1.**

**Table S9 Enriched KEGG pathway in LIGHT, TH, LTH, and UNREP genes in Experiment_1.**

**Table S10 Enriched gene ontology in DEGs between FL/FTH and other conditions in Experiment_2.**

**Table S11 Enriched gene ontology in LIGHT, TH, and LTH genes in Experiment_2.**

**Table S12 Enriched KEGG pathway in DEGs between FL/FTH and other conditions in Experiment_2.**

**Table S13 Enriched KEGG pathway in LIGHT, TH, and LTH genes in Experiment_2.**

**Table S14 Parameters of the gene expression model in Nagano et al. (2012) for genes related to photosynthetic light harvesting.**

**Table S15 Multiple comparison tests of starch and sucrose contents between conditions in Experiment_1.**

**Table S16 List of genes whose mean expression was higher in FIELD than in FL/FTH.**

**Table S17 List of genes whose mean expression was lower in FIELD than in FL/FTH.**

**Table S18 List of genes whose expression was higher in paddy fields than in growth chambers as well as in FIELD than in FL/FTH.**

**Table S19 List of genes whose expression was lower in paddy fields than in growth chambers as well as in FIELD than in FL/FTH.**
